# *Drynaria roosii*-derived exosome-like nanovesicles promote alveolar socket healing via activation of ITPR3-mediated calcium flux

**DOI:** 10.1186/s12951-026-04530-y

**Published:** 2026-05-20

**Authors:** Yueting Lin, Jiang Tao

**Affiliations:** Department of General Dentistry, Shanghai Ninth People’s Hospital, Shanghai Jiao Tong University School of Medicine, College of Stomatology, Shanghai Jiao Tong University, National Center for Stomatology, National Clinical Research Center for Oral Diseases, Shanghai Key Laboratory of Stomatology, Shanghai Research Institute of Stomatology, No. 500 Qu Xi Road, Shanghai, 200011 China

**Keywords:** Exosome-like nanovesicles, Alveolar socket healing, *Drynaria roosii*, Naringenin chalcone, ITPR3

## Abstract

Alveolar socket healing post-extraction is accompanied by alveolar bone loss, which is negative to subsequent prosthetic rehabilitation. Plant-derived exosome-like nanovesicles are extracellular vehicles with great potential for disease treatment and are advantageous for drug delivery and absorption. Herein, we report the mineralization-promoting effects of *Drynaria roosii*-derived exosome-like nanovesicles (DRDENs) on osteoblasts and in a mouse alveolar socket healing model. DRDENs were isolated and characterized. Treatment with DRDENs increased the formation of calcium nodules and the expression of osteogenic differentiation markers (ALP, RUNX2, and OCN) in osteoblasts. Compounds analysis of DRDENs revealed a previously unexplored component, naringenin chalcone. We evaluated the properties of naringenin chalcone and its bioactivity on osteoblast differentiation. In vivo studies confirmed the safety and efficacy of DRDENs and naringenin chalcone in bone regeneration. The mechanism was demonstrated in which DRDENs were endocytosed by cells, and naringenin chalcone was released to interact with the inositol 1,4,5-trisphosphate receptor type 3 (ITPR3) in the endoplasmic reticulum (ER), thereby activating downstream calcium flux to enhance bone formation. Additionally, chalcone isomerase (CHI), a key enzyme involved in naringenin chalcone production in *Drynaria roosii*, was purified and functionally identified as a type I CHI with weak II CHI activity. Our findings provided a novel strategy for alveolar socket healing and shed light on the function of CHI in *Drynaria roosii.*

**Graphical abstract:**

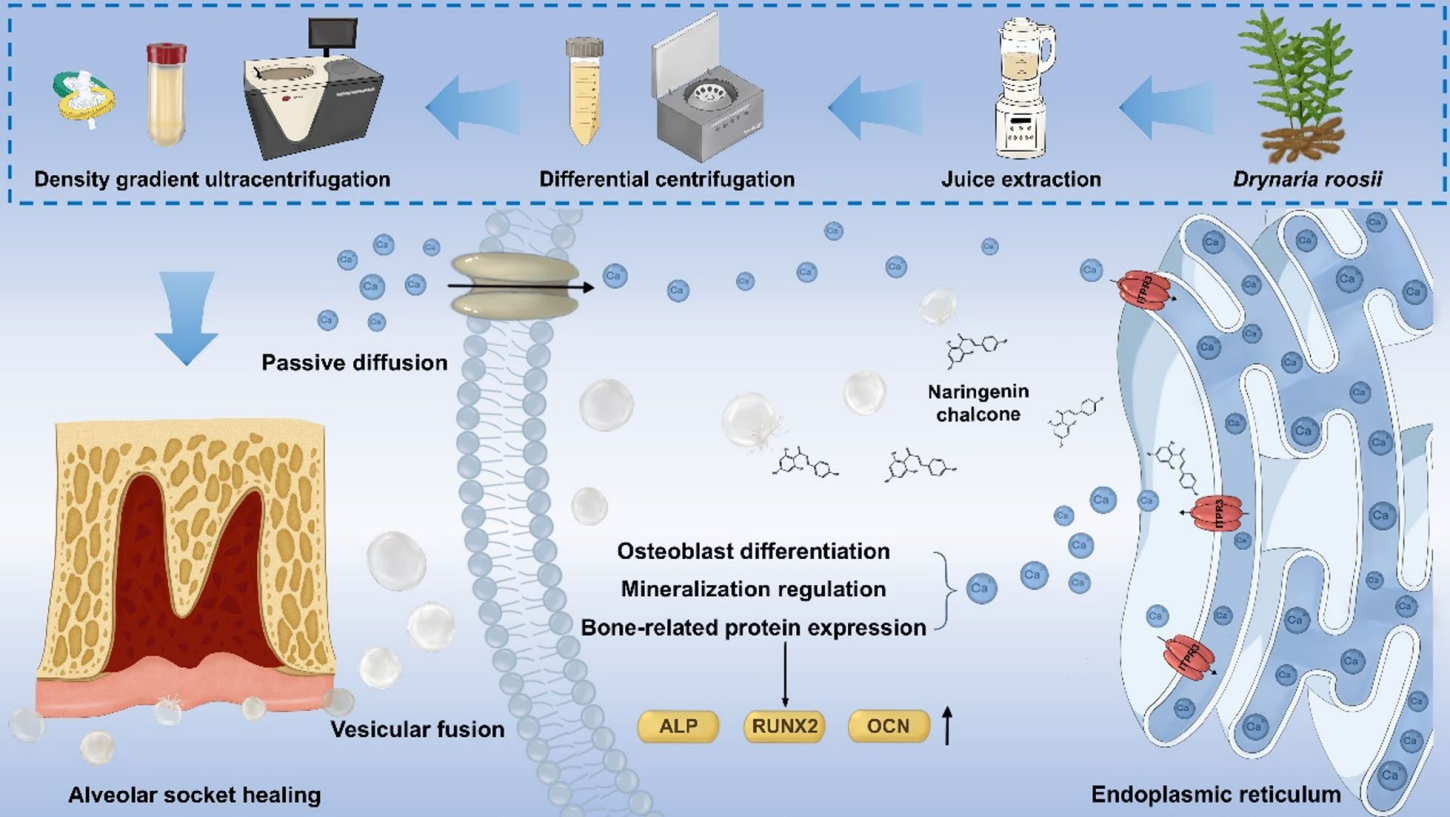

**Supplementary Information:**

The online version contains supplementary material available at 10.1186/s12951-026-04530-y.

## Introduction

The healing of the alveolar socket after tooth extraction is a complex process involving multiple cell types and signaling pathways [1, 2]. To close the extraction socket, surrounding tissues undergo marked horizontal and vertical contraction, progressing through four sequential stages: hemostasis/coagulation, inflammation, proliferation, and modeling/remodeling [3]. According to experimental and clinical reports, alveolar bone height can decrease by 29–63% in the horizontal dimension and 11–22% in the vertical dimension at 6 months post-extraction [4]. The significant alveolar bone reduction severely compromises subsequent dental implant placement or denture rehabilitation. Thus, safe and practical strategies that promote extraction socket healing and mitigate bone loss were urgently in demand.

Extracellular vesicles (EVs) are membrane-encapsulated particles produced by cells from microbes, plants, and animals, carrying bioactive molecules that mediate intercellular communication [5]. Exosomes are the smallest EVs, measuring 30–150 nm, and can be harvested from biological fluids and cell culture supernatants [6]. Accumulating studies have demonstrated their therapeutic potential in tissue regeneration, immunity modulation, and drug delivery [7–9].

Exosome-like nanovesicles from mammalian cells have been extensively studied and exhibit significant biological activities. However, isolation of exosomes from cultured cells in vitro is time-consuming and costly [10]. In contrast, plant-derived exosome-like nanovesicles are more economically viable, naturally abundant, and easier to harvest, making them attractive alternatives for biomedical applications. Recently, the therapeutic effects of plant-derived exosome-like nanovesicles have been reported. Lemon exosome-like nanoparticles were able to manipulate probiotics to prevent *Clostridioides difficile* infection [11]. Exosome-like nanoparticles from *Turmeric* exert therapeutic effects to alleviate ulcerative colitis [12]. Yam-derived exosome-like nanovesicles were shown to promote osteoblast differentiation in osteoporosis [13], and *Pueraria lobata* root-derived exosome-like nanovesicles have therapeutic effects on mitigating alcoholic intoxication by enhancing alcohol metabolism [14]. These findings indicate the immense therapeutic potential of plant-derived exosome-like nanovesicles.

*Drynaria roosii* is an edible and medicinal fern with year-round availability [15]. Flavonoid extracts from *Drynaria roosii* have been shown to promote bone reconstruction and prevent bone loss [16]. Previously identified active constituents in *Drynaria roosii* include naringenin, naringin, and others [17]. However, the therapeutic potential of *Drynaria roosii*-derived exosome-like nanovesicles with their bioactive components in alveolar socket healing remains uninvestigated.

In this study, we isolated and characterized exosome-like nanovesicles from *Drynaria roosii* and evaluated their cellular uptake, biocompatibility, and osteogenic activity in mouse pre-osteoblast MC3T3-E1 cells and human dental follicle stem cells (hDFSCs). We identified an unexplored and dominant bioactive component, naringenin chalcone, in DRDENs. The stability, cytotoxicity, and osteogenic effect of naringenin chalcone were systematically investigated. Using a mouse tooth extraction model, we confirmed the in vivo safety and efficacy of DRDENs and naringenin chalcone in promoting alveolar bone regeneration. Furthermore, we elucidated the molecular mechanism of DEDENs-mediated osteogenesis via naringenin chalcone. Additionally, the quantification of naringenin chalcone in DRDENs was calculated, and the chalcone isomerase in *Drynaria roosii*, which is involved with naringenin chalcone production, was purified and functionally identified.

## Methods

### Cell cultivation and identification

MC3T3-E1 cells were purchased from Fuheng Biotechnology (Shanghai, China). hDFSCs that are essential to periodontium development and possess the potential to differentiate into osteoblasts [18] were isolated from third molars of patients with informed consent and ethical approval (No.: SH9H-2025-T339-2). All cells were cultured in α-MEM medium (Gbico, USA) supplemented with 10% fetal bovine serum (Bioexplorer, USA) and 1% (v/v) penicillin-streptomycin solution, maintained at 37 °C in a humidified atmosphere with 5% CO_2_.

MC3T3-E1 cells were authenticated by short tandem repeat (STR) locus analysis at passage 1 and passage 18. For hDFSCs identification, surface antigens (CD34, CD105, CD71, CD90, and CD45) were analyzed by flow cytometry. hDFSCs used in multilineage differentiation potential detection (osteogenesis, adipogenesis, and chondrogenesis) were induced for 21 days, followed by staining with alizarin red S (OriCell, China), oil red O, and alcian blue (HyCyte, China), respectively.

### Isolation and purification of DRDENs

Fresh *Drynaria roosii* was collected from Jinhua, Zhejiang, China. The sarcocarp was peeled, homogenized using a blender (Midea, China), and filtered through a mesh to remove crude fibers. The resulting sap was subjected to sequential centrifugation to remove debris (3000 × g for 15 min, 10000 × g for 30 min at 4 °C; Hitachi, Japan). The supernatant was then ultracentrifuged at 100,000 × g for 60 min at 4 °C (Beckman, USA). The pellet containing nanovesicles was resuspended in PBS and purified by sucrose density gradient centrifugation (8%, 15%, 30%, 45%, and 60%) at 100,000 × g for 120 min at 4 °C. Fractions corresponding to the 30%-45% sucrose layer were collected, washed with 10× volume of PBS, and ultracentrifuged again at 100,000 × g for 60 min at 4 °C to remove sucrose. The concentration of DRDENs was quantified using a ZetaView NTA instrument (Particle Metrix, Germany).

### Characterization of DRDENs

DRDEN morphology was visualized by transmission electron microscopy (TEM; Hitachi, Japan). Briefly, freshly isolated DRDENs were adsorbed onto copper grids for 1 min, stained with 2% uranyl acetate for 3 min, and air-dried before imaging. Particle size distribution, concentration, and ζ-potential were analyzed using ZetaView NTA (Particle Metrix, Germany) and Zetasizer Nano ZS-90 (Malvern, UK).

### Alkaline phosphatase (ALP) staining and alizarin red S (ARS) staining

ALP and ARS staining were performed to assess early and late osteogenic differentiation, respectively. MC3T3-E1 cells and hDFSCs were seeded in 24-well plates. When cells reached 70% confluence, osteogenic induction medium (50 mg/L ascorbic acid and 10 mM β-glycerophosphate) was added. After 2 days, cells were treated with DRDENs or naringenin chalcone. On day 7, ALP staining was performed in the dark using an ALP staining kit (Beyotime, China) for 30 min. On day 21–28 post-induction, ARS staining was conducted according to the manufacturer’s instructions (OriCell, China). The quantitative analysis of ALP activity was performed using an alkaline phosphatase assay kit (Nanjing Jiancheng, China), and ARS quantification in each well was conducted by incubation with 10% cetylpyridinium chloride for 15 min, followed by absorbance reading at 562 nm with a microplate reader (Tecan, Switzerland).

### qRT-PCR analysis

qRT-PCR was used to assess mRNA levels of osteogenic-related genes. Total RNA of MC3T3-E1 cells and hDFSCs was extracted using a total RNA extraction kit (Vazyme, China) and reverse-transcribed into cDNA using a PrimeScript RT Reagent Kit (Takara, Japan). qRT-PCR was performed on a QuantStudio 6 Flex system (Thermo Fisher Scientific, USA) using SYBR Green Master Mix (Vazyme, China). Target genes included osteogenic markers ALP, Runt-related transcription factor 2 (RUNX2), and osteocalcin (OCN), with β-actin as the internal reference. Primer sequences are listed in Supplementary Table S1. Relative mRNA levels were calculated using the 2^−ΔΔCt^ method.

### Cellular uptake of DRDENs

DRDENs were labeled with PKH26 (Solarbio, China) according to the manufacturer’s protocol. PKH26-labeled DRDENs (1 × 10^8^ particles/mL) were co-incubated with MC3T3-E1 cells and hDFSCs for 6–8 h. The medium was discarded, and cells were stained with Calcein-AM (1 µM) for 1 h at 37 °C to visualize the cytoplasm. Cells were fixed with 4% paraformaldehyde for 20 min and stained with DAPI for 5 min to label nuclei. After washing with PBS, cellular uptake of DRDENs was observed using a laser scanning confocal microscope (Leica, Germany).

### Cell proliferation assay

Cell viability was evaluated using the Cell Counting Kit-8 (CCK-8; Yeasen, China). MC3T3-E1 cells and hDFSCs were seeded in 96-well plates at a density of 5 × 10^3^ and 1 × 10^4^ cells/well. After 48 h, cells were treated with different concentrations of DRDENs or naringenin chalcone. At specified time points, 10% CCK-8 solution was added to each well, and cells were incubated for 1 h at 37 °C in the dark. Absorbance at 450 nm was measured using a microplate reader (Thermo Multiskan Sky, USA).

### Hemolytic test

Mouse erythrocytes were prepared as a 4% (v/v) suspension in PBS. Aliquots of 1 mL erythrocyte suspension were centrifuged at 3000 rpm for 10 min, and the supernatant was discarded. The pellet was resuspended in 1 mL of DRDENs or naringenin chalcone solution at various concentrations. PBS served as the negative control for DRDENs group, isotonic saline as the negative control for naringenin chalcone group, and ddH_2_O as the positive control. The mixtures were incubated at 37 °C for 3 h, then centrifuged at 3000 rpm for 10 min. The absorbance of the supernatant was measured at 540 nm using a microplate reader. Hemolytic rate was calculated as follows: [(A_540_ of test sample – A_540_ of negative control) / (A_540_ of positive control – A_540_ of negative control)] × 100%.

### Compounds analysis of DRDENs

DRDENs solution was mixed with twice the volume of acetonitrile, and centrifuged at 8000 rpm for 10 min. The supernatant was filtered through a 0.22 μm membrane before analysis. Compounds analysis of DRDENs was conducted using a Thermo Fisher UPLC-MS/MS system (Thermo Fisher, USA) with an ACQUITY UPLC BEH C18 column (2.1 mm x 100 mm, 1.7 μm, Waters).

A 30-minute elution method was used at a flow rate of 0.3 mL/min. Mobile phase A consisted of water and 0.1% (v/v) formic acid. Mobile phase B consisted of acetonitrile and 0.1% (v/v) formic acid. DRDENs were eluted with a linear gradient from 5% B for 1 min, 5% to 40% B for 3 min, 40% to 80% B for 11 min, 80% to 95% B for 8 min, 95% B for 4 min, and equilibrated with 5% B for 3 min. The mass spectrometer was set to electrospray ionization in both positive and negative ion modes.

### Fingerprint analysis of the DRDENs

Fingerprint analysis was performed on an Agilent LC-MS system using a Poroshell 120 EC-C18 column (3.0 × 50 mm, 2.7 μm, Agilent). Water with 0.1% (v/v) formic acid (A) and acetonitrile with 0.1% (v/v) formic acid (B) were used as mobile phase at a flow rate of 0.5 mL/min. Seven batches of DRDENs, naringenin chalcone standard, and naringenin standard were analyzed using the following gradient: 40% to 95% (v/v) B for 3 min, 95% B for 1 min, and equilibrated with 40% B for 3 min. UV absorption was recorded at 375 nm.

### Biodistribution of DRDENs

DRDENs were labeled with DiR and administered to mice via tail vein injection, intraperitoneal injection, or oral gavage. In vivo imaging was performed at 2, 6, 12, 24, and 48 h using an in vivo imaging system (IVIS; PerkinElmer, USA). After 48 h, mice were euthanized, and major organs (heart, liver, spleen, lung, kidney, gastrointestinal tract) and related tissues (cranio-maxillary bone, mandible, tongue, tibia) were harvested for ex vivo fluorescence imaging.

### Stability test of naringenin chalcone

HPLC analysis of naringenin chalcone was performed on an Agilent HPLC 1260 series system using a ShimNex CS C18 column (250 mm × 4.6 mm, 5 μm, Shimadzu). Water with 0.1% (v/v) formic acid (A) and acetonitrile (B) was used as the mobile phase at a flow rate of 0.8 mL/min. The elution gradient was as follows: 10% to 30% (v/v) B for 15 min, 30% B for 15 min, 30 to 100% B for 10 min, washed with 100% (v/v) solvent B for 5 min, and equilibrated with 10% solvent B for 5 min [19]. UV absorption was all recorded.

For pH stability analysis, 1 mM naringenin chalcone was prepared in ethanol solutions with a pH gradient (adjusted with 1 M NaOH) in 96-well plates. Full-wavelength scanning (230–1000 nm) and kinetic measurements at 375 nm for 60 min were performed using a SpectraMax iD5 microplate reader (Molecular Devices, USA). To evaluate interactions with serum albumin, naringenin chalcone (100–2000 µM) was dissolved in 1% DMSO/30% PEG300/69% isotonic saline and incubated with 5 mg/mL bovine serum albumin (BSA). Full-wavelength spectra were recorded at 10 and 60 min.

### Construction of the tooth extraction model and the treatment

Animal experiments were approved by the Laboratory Animal Ethics Committee of the Shanghai Ninth People’s Hospital, Shanghai Jiao Tong University School of Medicine (Approval No.: SH9H-2025-A1638-1). Four-week-old C57BL/6 mice were purchased from Shanghai Jihui Biotechnology Co., Ltd. After 2 weeks of adaptive feeding, mice were randomly divided into 6 groups (*n* = 9 per group). The maxillary first molar (M1) was extracted under general anesthesia. DRDENs (5 × 10^11^ particles/kg per mouse) [20] or naringenin chalcone (20 mg/kg per mouse) [21], based on previous studies, were orally administered every 2 days.

### In vitro digestion assay

DRDENs were incubated in a simulated gastric buffer with slow rotation at 37 °C for 1 h [22]. Then, the TEM was used to observe the morphology of digested DRDENs, and the size distribution and concentration were determined with a ZetaView device (Particle Metrix, Germany).

### Histological staining

At 14 and 28 days post-treatment, mice were euthanized. Major organs (heart, liver, spleen, lung, kidney) were fixed in 4% paraformaldehyde for 24 h, dehydrated with gradient ethanol, embedded in paraffin, and sectioned (5 μm). Sections were stained with hematoxylin and eosin (H&E) and Masson’s trichrome dye for histological evaluation.

Maxillary bones were decalcified in 10% EDTA for 2 weeks, then embedded in paraffin. Section (5 μm) were subjected to H&E staining, Masson’s trichrome staining, and immunohistochemical (IHC) staining. For IHC staining, sections were dewaxed, rehydrated, and subjected to antigen retrieval. Endogenous peroxidase activity was blocked by incubation with 3% H_2_O_2_ for 10 min. Sections were blocked with 5% BSA for 1 h at room temperature, then incubated with primary antibodies against RUNX2 and OCN (ABclonal, China) at 4 °C overnight. After washing with PBS, sections were incubated with an HRP-conjugated secondary antibody for 1 h at room temperature, visualized with diaminobenzidine, and counterstained with hematoxylin. Sections were dehydrated, cleared, and sealed with neutral resin before imaging. Histological staining was quantified using ImageJ software.

### Micro-CT analysis

Maxillary bones were harvested after euthanasia, fixed in 4% paraformaldehyde for 24–48 h, and scanned using a Zeiss Xradia MicroXCT-200 system (Zeiss, Germany) with 1000 slices per sample. Three-dimensional reconstruction and quantitative analysis, including relative bone volume/total volume (BV/TV) and bone mineral density (BMD), were performed using Dragonfly.

### Enzyme-linked immunosorbent assay

At 14 and 28 days post-treatment, mouse blood was collected and allowed to clot at 4 °C for 60 min. Serum was separated by centrifugation at 2000 rpm for 15 min. Concentrations of procollagen I N-terminal propeptide (PINP) and tartrate-resistant acid phosphatase-5b (TRACP-5b) were measured using commercial ELISA kits (Elabscience, China) according to the manufacturer’s instructions.

### RNA-sequencing analysis

At 14 days post-treatment, the extraction socket region of the maxillary first molar was dissected, and total RNA was extracted. RNA sequencing was performed by Hangzhou Guangkeande Biotechnology Co., Ltd. Raw data were processed through quality control, data cleaning, gene annotation, genome alignment, differential expression analysis, and Gene Ontology (GO) enrichment analysis.

### Molecular docking and molecular dynamics simulation

The 3D structure of human ITPR3 protein (Identifier: 8tk8) was downloaded from UniProt (https://www.uniprot.org/), and the structure of naringenin chalcone was downloaded from PubChem (https://pubchem.ncbi.nlm.nih.gov/) and energy-minimized using the MMFF94 force field. Molecule docking was performed using AutoDock Vina 1.1.2 [23], and the conformation with the highest binding score was selected for further analysis. Molecular dynamics simulation of naringenin chalcone and ITPR3 was performed using Amber 18 [24] under constant pressure and temperature conditions for 100 ns. Root mean square deviation (RMSD), root mean square fluctuation (RMSF), radius of gyration (RoG), solvent-accessible surface area (SASA), and molecular mechanics/generalized born surface area (MM/GBSA) were calculated to evaluate the stability of the complex.

### Surface plasmon resonance

Surface plasmon resonance (SPR) provided a sensitive, real-time, and label-free technique to monitor the interaction between the ligand and the receptor [25]. Recombinant human ITPR3 fragment (2472–2582 aa; Ipodix, China) was immobilized on a CM5 sensor chip (Cytiva, USA) using an amine-coupling kit according to the manufacturer’s instructions (Cytiva, USA). Naringenin chalcone was prepared at concentrations ranging from 0.3125 to 10 µM, and the solutions were served as a running buffer that flowed over the chip from low to high concentration at 30 µL/min for 60 s. Binding kinetics were analyzed using a Biacore Insight system (Cytiva V6.0, Marborough, MA, USA) and fitted to a 1:1 (Langmuir) binding model. The K_D_ value was calculated using the formula K_D_ =k_d_/k_a_, where k_d_ is the dissociation rate constant and k_a_ is the association rate constant.

### Point mutation of ITPR3

Amino acids residues of ITPR3 with binding free energy (ΔG_bind_) exceeding −1 kcal/mol from MM/GBSA analysis were subjected to computational alanine scanning using FoldX 5.0 [26]. Residues with ΔΔG_bind_ greater than 0.5 kcal/mol were selected for saturated mutagenesis. The CRISPR-Cas9 technique was used to introduce point mutations (L2523Y and E2526R) into ITPR3 in hDFSCs. Lentiviral plasmid (lenti-U6-ITPR3(human)-sgRNA-Cas9-EGFP) and donor RNA plasmid (AAV-ITPR3-Donor-mCherry-puro) were constructed and packaged in HEK293T cells. After the transfection and antibiotic selection, mutated cells were verified by DNA sequencing. Primer sequences are listed in Supplementary Table S2.

### Western blot

Cells were lysed with RIPA solution containing protease inhibitor for 30 min on ice. Protein in the supernatant was extracted after centrifuging at 12,000 × g for 10 min at 4 °C, and protein concentration was determined using a BCA Protein Assay Kit (Yeasen, China). The proteins were separated by SDS-PAGE, transferred to PVDF membranes (Millipore, USA), and blocked with 5% skim milk. Subsequently, the membranes were incubated with primary antibodies against ALP, runt-related transcription factor 2 (RUNX2), osteocalcin (OCN) (ABclonal, China), and β-Actin (Abmart, China) at 4 °C overnight. After washing with TBST, membranes were incubated with HRP-conjugated secondary antibody for 1 h at room temperature. Ultimately, the proteins were detected using a chemiluminescence image analysis system (UVItec, Britain) with enhanced chemiluminescent (ECL) solution.

### Measurement of Ca^2+^ fluxion in the endoplasmic reticulum

Mag-Fluo-4 AM was employed to trace Ca^2+^ flux in the endoplasmic reticulum, as previously described [27]. Cells were adherent cultured in a 96-well black-walled plate for 2–3 d, and incubated with 10 µM Mag-Fluo-4 AM for 30 to 60 min at 37 °C in saline buffer (135 mM NaCl, 5.9 mM KCl, 11.6 mM HEPES, 1.5 mM CaCl_2_, 11.5 mM glucose, 1.2 mM MgCl_2_, pH 7.3) before permeabilizing with 10 µg/mL saponin (37 °C, 3–5 min) in Ca^2+^ free cytosol-like medium (20 mM NaCl, 140 mM KCl, 1 mM EGTA, 20 mM PIPES, 2 mM MgCl_2_, pH 7.0). The Mg^2+^-free cytosol-like medium, supplemented with 220 nM CaCl_2_ and a series of naringenin chalcone solutions, was then added. Fluorescence intensity (excitation at 485 nm, emission at 535 nm) was recorded continuously for 15 min using a microplate reader (Tecan, Switzerland).

### Quantitative analysis of naringenin chalcone

To determine the concentration of naringenin chalcone in DRDENs, a standard curve of naringenin chalcone was established with 12.5, 25, 50, 100, 200, and 400 µM naringenin chalcone standard solutions. 2 µL of each naringenin chalcone solution was injected into the LC-MS at 375 nm absorbance. The corresponding molar mass of naringenin chalcone was calculated as 25–800 pmol on the horizontal axis. Peak areas for each concentration were used as vertical coordinates. The concentration of naringenin chalcone was then computed using the regression equation from the standard curve. The LC-MS elution method was as mentioned in the fingerprint analysis section. 

### Gene cloning and plasmid construction of CHI

The sequence of *Drynaria roosii* chalcone isomerase (DrCHI) was mined from NCBI Sequence Read Archive (SRA: SRS2253291). We downloaded the raw sequences of the entire *Drynaria roosii* transcriptome to establish a local database. Using several known CHIs of ferns, the CHI sequence of *Drynaria roosii* was thereby spliced and deduced. Total RNA from fresh *Drynaria roosii* was extracted using a plant RNA extraction kit (Simgen, China) and reverse-transcribed into cDNA or gDNA. Afterward, *DrCHI* fragments were amplified by PCR using specific primers (Supplementary Table S2) and cloned into the pET28a vector after digestion with EcoRI and XhoI. Recombinant plasmids were transformed into *E. coli* DH5α for amplification and sequencing verification.

### Multiple sequence alignment and phylogenetic analysis

Multiple sequence alignment of DrCHI with other 22 pteridophyte CHIs was performed using R 4.1.3, and a phylogenetic tree was constructed using MEGA 7.0 and ITOL (http://itol.embl.de/) [28].

### Expression and purification of drynaria roosii CHI

For the expression of DrCHI, recombinant plasmids were transformed into *E. coli* Rosetta. Furthermore, the cells carrying the recombinant plasmids were cultivated in LB broth containing 100 µg/mL kanamycin at 37 °C until the OD_600_ reached 0.6. IPTG (0.5 mM) was then added to induce DrCHI expression at 16 °C for 16–18 h. After centrifugation at 4000 rpm for 10 min, lysis buffer (50 mM NaH_2_PO_4_, 300 mM NaCl, 10 mM imidazole) was added to resuspend the cell pellets. Then the cells were disrupted by ultrasonication on ice, and were centrifuged (4000 rpm, 4 °C for 30 min). The supernatant was loaded onto a Ni-NTA agarose column, and the protein was eluted with increasing imidazole concentrations (40–250 mM). Before being concentrated, the eluent was desalted using a desalting column with the exchange buffer (50 mM Tris, 10 mM NaCl, 10% glycerol). The concentration of purified protein was determined using a microspectrophotometer (Nano 500, China). Purity of the protein was confirmed by SDS-PAGE.

### In vitro enzyme assay of drynaria roosii CHI

The enzyme activity of DrCHI was assayed in a 100 µL reaction mixture containing 50 mM Tris-HCl buffer (pH 7.5), 2 mM DTT, 100 µM substrate (naringenin chalcone or isoliquiritigenin), and 10 µg purified DrCHI. The reaction was incubated at 25 °C for 1 min and terminated by adding 200 µL methanol. The mixture was filtered through a 0.22 μm membrane and analyzed by LC-MS. The elution method was described in the fingerprint analysis above. UV absorption was recorded at 280 nm.

### Statistical analysis

Data are presented as mean ± standard deviation (SD) from at least three independent experiments. Statistical analysis was performed using Student’s *t*-test or one-way ANOVA. Statistical significance was defined as *p* < 0.05 *, *p* < 0.01 **, and *p* < 0.001 ***.

## Results

### Physicochemical properties and fingerprint of DRDENs

**Fig. 1:**
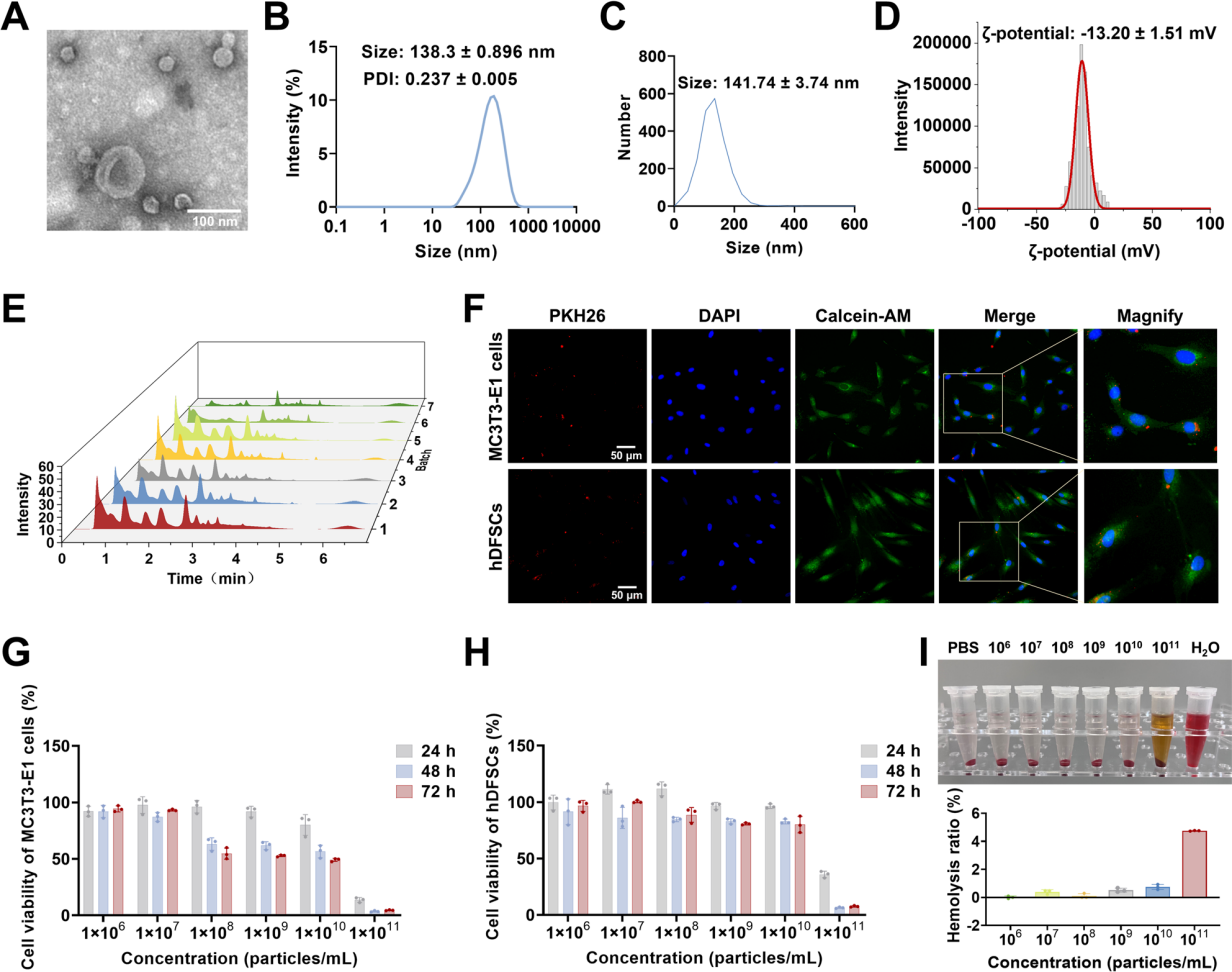
Characterization and biocompatibility of DRDENs. (**A**) TEM imaging of DRDENs. (**B**-**C**) Size distribution measured by NTA and DLS. (**D**) ζ-potential histogram. (**E**) HPLC fingerprint chromatography. (**F**) Cellular uptake of PKH26-labeled DRDENs in MC3T3-E1 cells and hDFSCs (20×). (**G**-**H**) Cell viability of MC3T3-E1 cells and hDFSCs after incubating with DRDENs for 24, 48, and 72 h. (**I**) Hemolysis experiment of DRDENs

DRDENs were successfully isolated from fresh *Drynaria roosii* juice via differential centrifugation and sucrose density gradient ultracentrifugation. TEM image revealed typical spherical exosome-like morphology (Fig. 1A). Dynamic light scattering (DLS) analysis showed a uniform particle size distribution (Polydispersity Index, PDI = 0.237 ± 0.005) with an average diameter of 138.3 ± 0.896 nm (Fig. 1B), which was broadly consistent with nanoparticle tracking analysis (NTA) results of 141.74 ± 3.74 nm (Fig. 1C). The ζ- potential of DRDENs was − 13.2 ± 1.51 mV (Fig. 1D), indicating a negatively charged surface similar to that of healthy human platelets [29]. HPLC fingerprint analysis of seven DRDEN batches showed consistent chromatographic profiles, confirming good reproducibility (Fig. 1E).

### Cellular uptake and in vitro biocompatibility of DRDENs

MC3T3-E1 cells and hDFSCs were prepared for the following cell experiments. We checked the STR profiles of MC3T3-E1 cells at passages 1 and 18, confirming their correctness and genetic consistency (Supplementary Figure S1a, b). Identification of hDFSCs was examined by flow cytometry for positive expression of mesenchymal stem cell markers (CD73, CD90, CD105, CD31) and negative expression of endothelial/hematopoietic markers (CD45) (Supplementary Figure S2a), with confirmed multidirectional differentiation potential (Supplementary Figure S2b).

Cellular uptake is a critical mechanism by which nanovesicles deliver drugs and exert biological effects. We labeled DRDENs with the red PKH26 dye. As shown in Fig. 1F, DRDENs were efficiently internalized by MC3T3-E1 cells and hDFSCs, localizing in the cytoplasm.

CCK-8 assays demonstrated that DRDENs had no significant cytotoxicity at concentrations up to 1 × 10^10^ particles/mL in 24 h, with cell viability > 80% for both cell types at 24 h (Fig. 1G, H). The hemolytic test was used to evaluate the blood compatibility of DRDENs. It was shown that DRDENs did not induce hemolysis at concentrations ranging from 1 × 10^6^ to 1 × 10^11^ particles/mL (hemolytic rate < 5%) and exhibited good blood compatibility (Fig. 1I).

### In vivo distribution of DRDENs

**Fig. 2:**
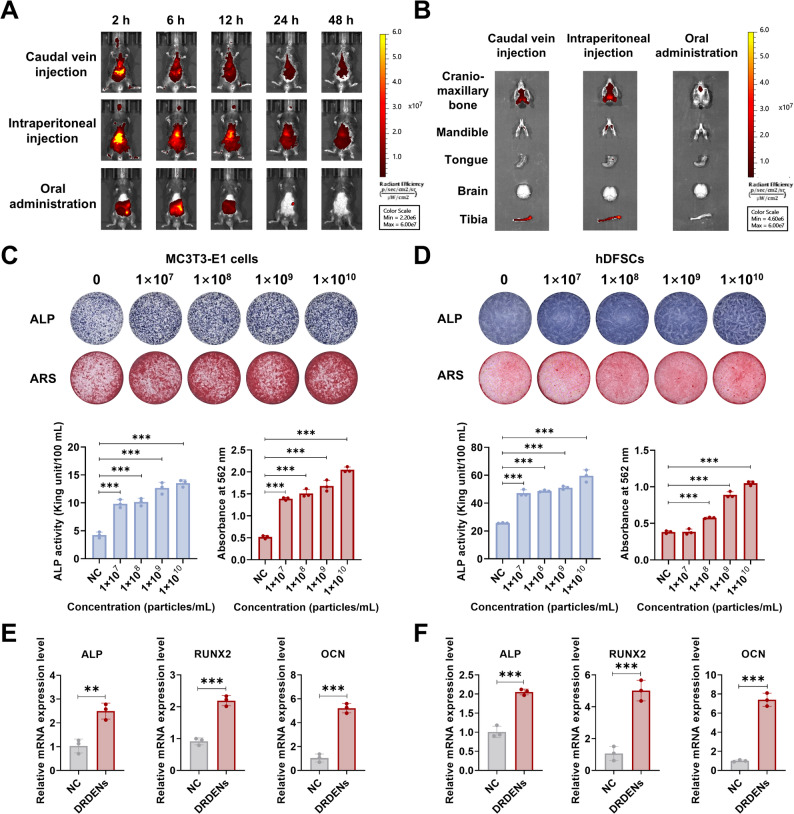
In vivo imaging and in vitro osteogenic activity of DRDENs. (**A**) Whole body imaging of the mice at 2, 6, 12, 24, and 48 h after administration of DiR-labeled DRDENs. (**B**) Distribution of DiR-labeled DRDENs in cranio-maxillary bone, mandible, tongue, brain, and tibia at 48 h. (**C**-**D**) Alkaline phosphatase activity at day 7 and alizarin red S staining at day 21-28 after treatment with different concentrations of DRDENs in MC3T3-E1 cells and hDFSCs, and the corresponding quantitative analysis. (**E**-**F**) ALP, RUNX2, and OCN mRNA expression levels in DRDENs-incubated MC3T3-E1 cells and hDFSCs were determined by qRT-PCR

Drug administration by different routes affects their absorption efficiency. Intravenous and intraperitoneal administration are more bioavailable than oral administration, while oral administration is the most acceptable and noninvasive option for patients. Different approaches may show different distributions. Therefore, we investigated the distribution of DRDENs after intravenous, intraperitoneal, and oral administration in mice using an IVIS imaging system. The DRDENs were labeled with the DiR fluorescent dye and administered to mice via caudal vein, intraperitoneal, and oral routes. As shown in Fig. 2A, DiR-labeled DRDENs reached peak fluorescence intensity at 6 h post-administration in all groups, followed by a gradual decline. Consistent with previous studies, the absorption after intravenous administration was less extensive than that after intraperitoneal injection [30]. Subsequently, the mice were euthanized, and the internal organs were removed 48 h post-administration for ex vivo imaging. It was revealed that DRDENs were mainly distributed in the abdomen in the intravenous and intraperitoneal administration groups, while no fluorescence was observed in the organs of the oral administration group (Supplementary Figure S3). Notably, DRDENs were detected in the cranio-maxillary bone of all three administration groups (Fig. 2B), suggesting that DRDENs could exert their function in the maxillary bone.

### In vitro osteogenesis effect of the DRDENs

To explore the osteogenic effects of DRDENs, ALP and ARS staining were used to assess osteoblast activity and mineralization. ALP is a critical bone-metabolism factor secreted early by osteoblasts. The presence of ALP could enhance extracellular matrix mineralization. ARS is a specific calcium-labeling dye used to assess late-stage mineralization [31]. We conducted ALP staining at 7 d and ARS staining at 21–28 d after DRDENs were added. As shown in Fig. 2C and D, DRDENs promoted osteogenic differentiation in a concentration-dependent manner.

Moreover, the levels of osteogenesis-related genes, including ALP, RUNX2, and OCN, were measured by qRT-PCR. RUNX2 is a core transcription factor associated with early-stage osteoblast differentiation. OCN is a marker produced by mature osteoblasts in the later period and would regulate the mineralization of bone matrix [32]. We chose 1 × 10^10^ particles/mL as the DRDENs dosing concentration to assess the osteogenesis-related gene expression levels. As shown in the figures, DRDENs significantly upregulated the mRNA expression of ALP, RUNX2, and OCN in MC3T3-E1 cells and hDFSCs (Fig. 2E, F), indicating enhanced early and late osteogenic differentiation.

### Compounds analysis of DRDENs

**Fig. 3:**
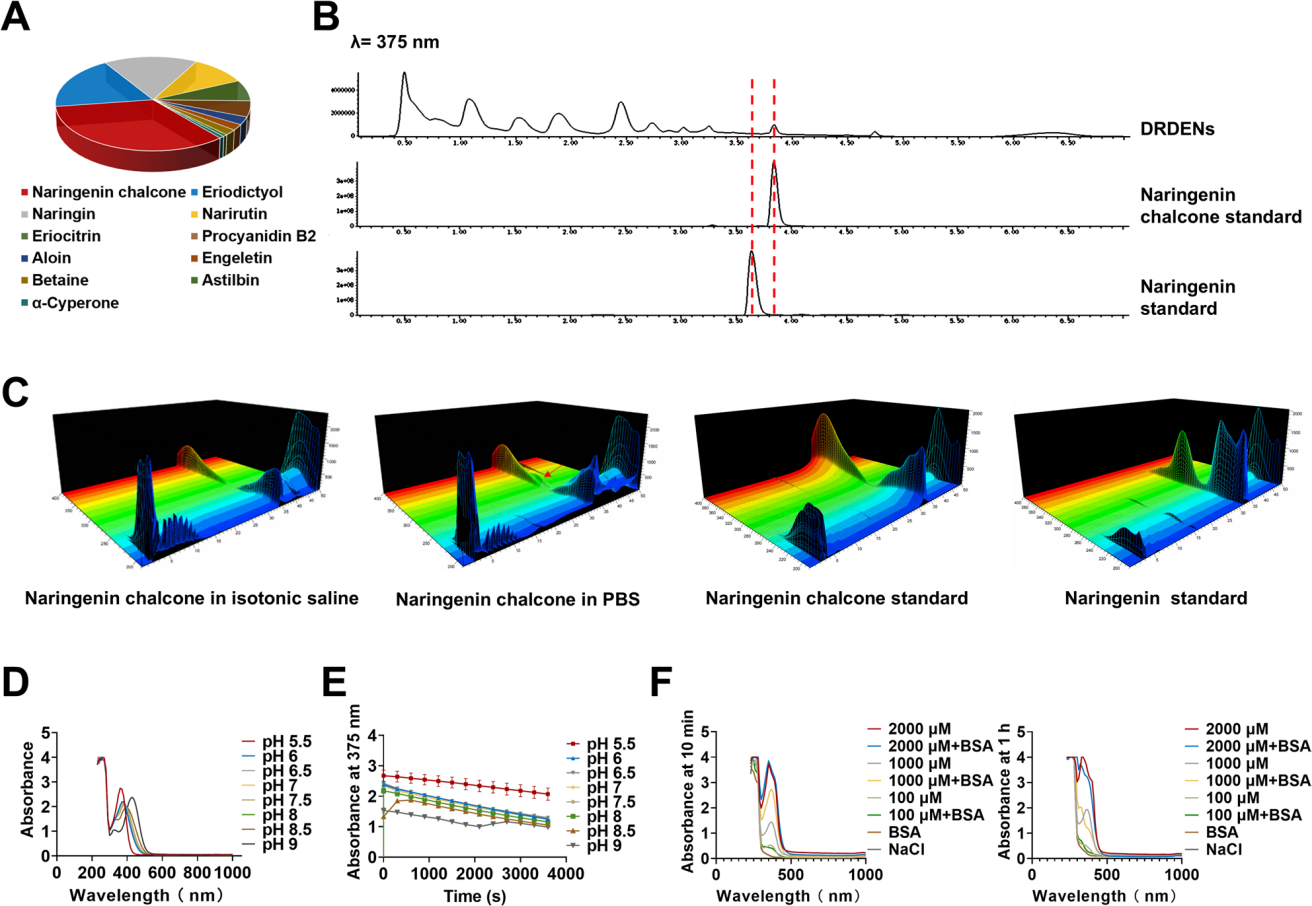
Properties of naringenin chalcone. (**A**) Molecular compositional analysis of DRDENs (**B**) Identification of naringenin chalcone in DRDENs by LC-MS. (**C**) HPLC analysis and comparison of naringenin chalcone in isotonic saline, and PBS (red arrow in the PBS group indicates the newly transformed naringenin). (**D**) Full-spectrum scanning to reveal the lability of naringenin chalcone under different pH conditions. (**E**) Kinetic measurement of naringenin chalcone for 60 min in its specific ultraviolet absorbance at 375 nm. (**F**) Incubation of naringenin chalcone with 5 mg/mL BSA for 10 min and 1 h

Plant-derived exosome-like nanovesicles are a delivery system with a membrane that packages the metabolic products plants produce. Thus, we investigated the compounds in DRDENs using UPLC-MS/MS. Several chemicals, including naringenin chalcone, eriodictyol, naringin, narirutin, etc., were found (Fig. 3A). Among these compounds, naringenin chalcone is an unexplored molecule in *Drynaria roosii*. Since naringenin chalcone had the highest area and peak rating in mass spectrometry, suggesting it as the dominant component in DRDENs, we set about investigating it.

### Identification and stability of naringenin chalcone

It may be ambiguous between naringenin chalcone and naringenin, as they have similar molecular weights of 272. Hence, we injected the DRDENs, naringenin chalcone standard, and naringenin standard solution into the LC-MS, respectively. By mass spectrometry analysis, we confirm that the molecule with a molecular weight of 272 in DRDENs is naringenin chalcone (Fig. 3B).

Notably, the naringenin chalcone was labile in solution. To enhance the understanding of naringenin chalcone and facilitate subsequent solution preparation. We conduct several experiments to investigate its stability. It was shown that naringenin chalcone was unstable in PBS (pH 7.4), converting to naringenin within 3 min, while it remained stable in isotonic saline (Fig. 3C).

Furthermore, we found that the color of the naringenin chalcone solution was lighter when an alkaline liquid was added or when it was left to stand for a while. These phenomena suggest that the changes in pH and time may compromise its stability. We then prepared naringenin chalcone solutions and adjusted their pH to a gradient in a 96-well plate. Full-wavelength scanning revealed a maximum UV absorption at 375 nm and a native pH of ~ 5.5 (Fig. 3D). Naringenin chalcone degradation was intense at pH > 8.5 (Fig. 3D, E).

As a previous study reported, naringenin chalcone is relatively stable in the presence of 5–10 mg/mL serum albumin [33]. We co-incubated naringenin chalcone with 5 mg/mL bovine serum albumin (BSA), and it is shown that the incubation with BSA enhanced absorbance and induced a blue shift (Fig. 3F), indicating interactions with serum albumin.

**Fig. 4:**
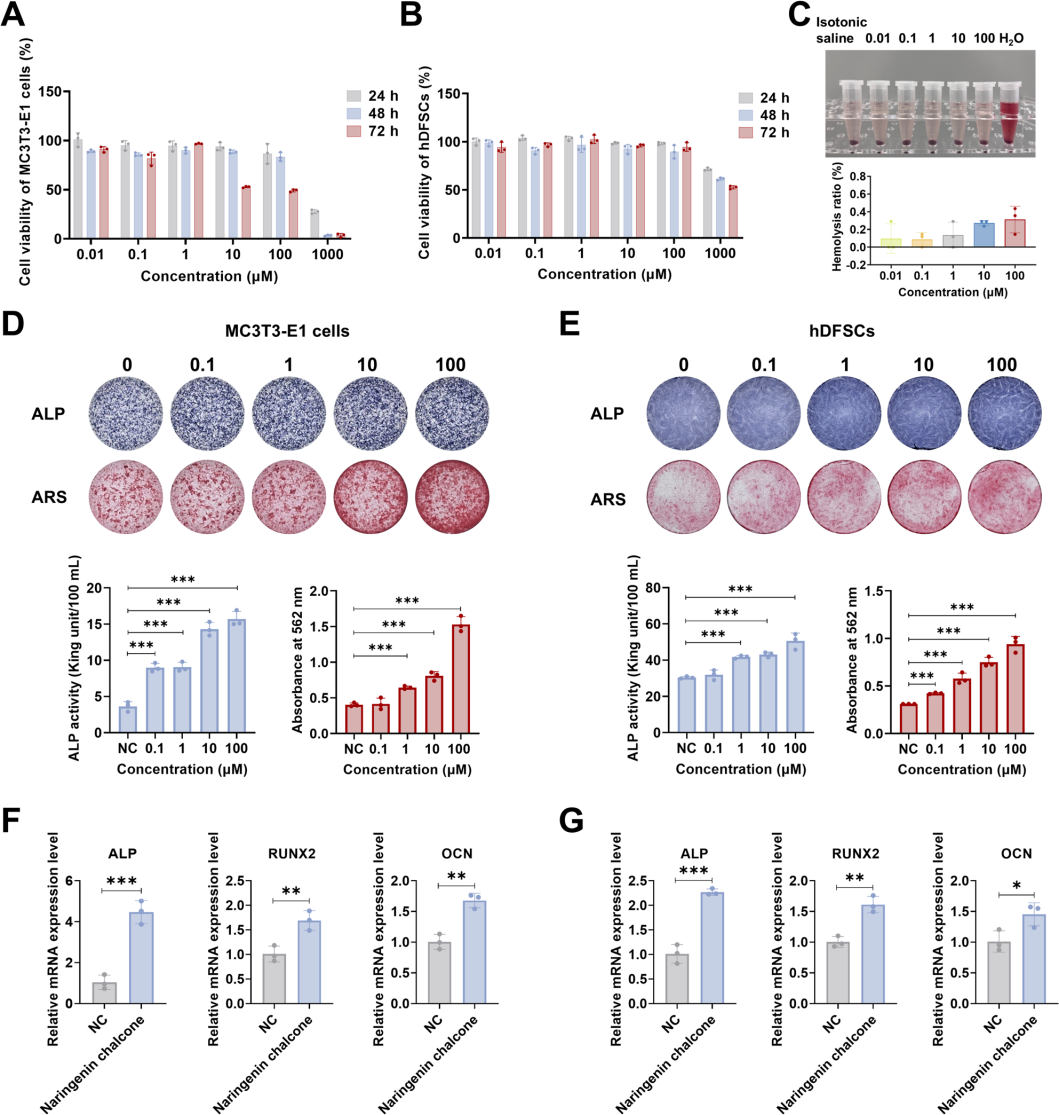
In vitro cytotoxicity and osteogenic activity of naringenin chalcone. (**A**-**B**) Cell viability of naringenin chalcone-treated MC3T3-E1 cells and hDFSCs in 24, 48, and 72 h. (**C**) Hemolysis experiment of naringenin chalcone. (**D**-**E**) Alkaline phosphatase activity and at day 7 and alizarin red S staining at day 21-28 after treatment with different concentrations of naringenin chalcone in MC3T3-E1 cells and hDFSCs, and the corresponding quantitative analysis. (**F**-**G**) ALP, RUNX2, and OCN mRNA expression levels in naringenin chalcone-treated MC3T3-E1 cells and hDFSCs, analyzed by qRT-PCR

### In vitro biocompatibility and osteogenic activity of naringenin chalcone

CCK-8 assays showed that naringenin chalcone (0.01–100 µM) had no significant cytotoxicity on MC3T3-E1 cells and hDFSCs (Fig. 4A, B). Hemolytic rates were < 0.5% for all concentrations tested (Fig. 4C), confirming in vitro safety.

ALP and ARS staining showed that naringenin chalcone promoted osteogenic differentiation in a concentration-dependent manner, with maximum activity at 100 µM (Fig. 4D, E). qRT-PCR analysis confirmed that 100 µM naringenin chalcone significantly upregulated the mRNA expression of ALP, RUNX2, and OCN in both cell types (Fig. 4F, G). These results indicated that naringenin chalcone in DRDENs shares similar osteogenic effects in vitro with DRDENs.

### In vivo safety and efficacy of the DRDENs and naringenin chalcone in the tooth extraction mouse model

**Fig. 5:**
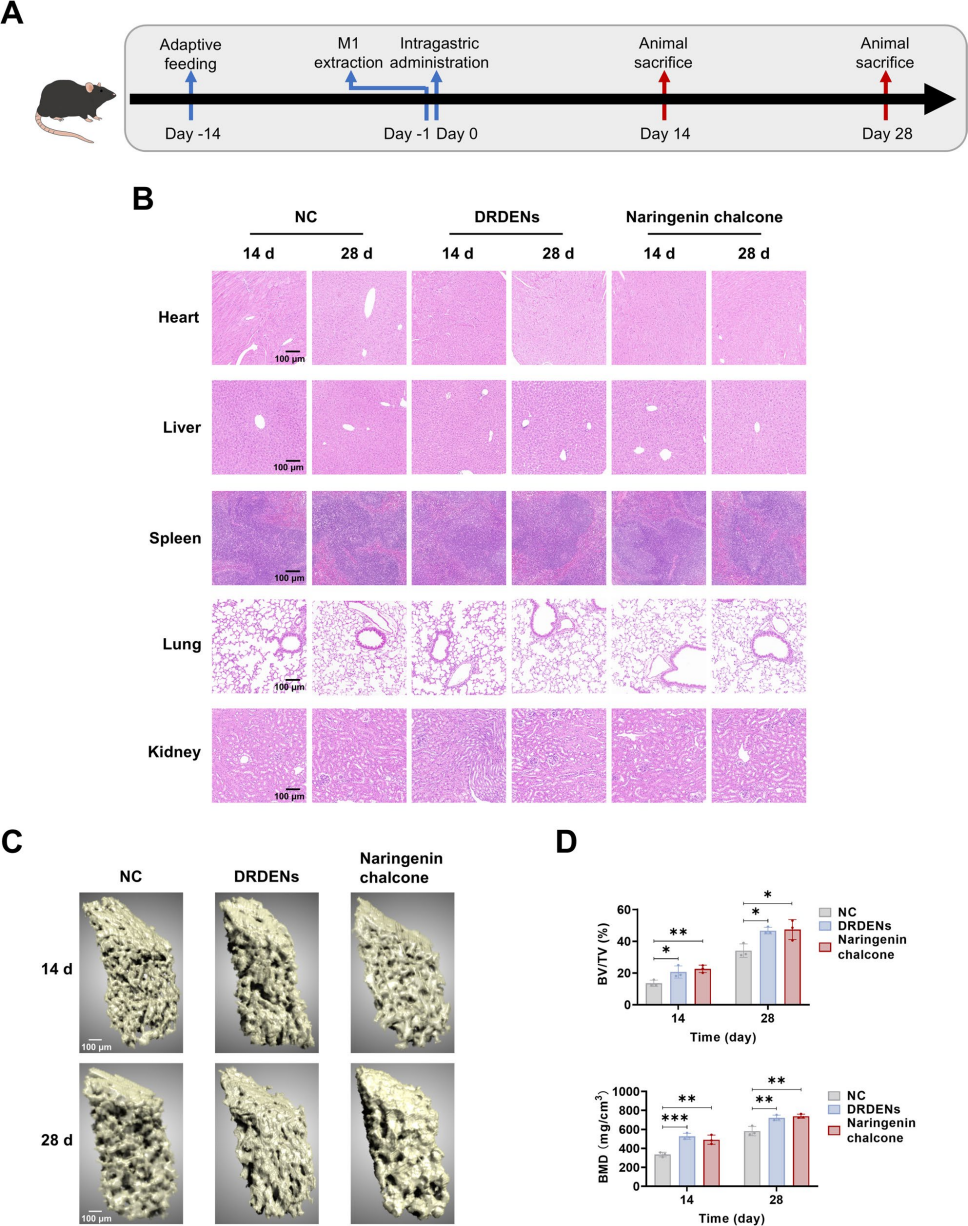
Osteogenic effects and safety evaluation of DRDENs and naringenin chalcone in tooth extraction mice. (**A**) Schematic diagram of maxillary first molar (M1) extraction mouse model subjected to DRDENs and naringenin chalcone treatment. (**B**) H&E staining of main organs slices from mice treated with DRDENs and naringenin chalcone (Scale bar = 100 μm). (**C**) Representative images of micro-CT analysis of the structure of the maxillary first molar alveolar socket (Scale bar = 100 μm). (**D**) Quantification of bone volume fraction (BV/TV) and bone mineral density (BMD) in M1 alveolar socket

Now that DRDENs and naringenin chalcone have been experimentally shown to be safe and to promote osteogenesis in vitro, a mouse tooth extraction model was developed to evaluate their in vivo effects (Fig. 5A). H&E staining of major organs (heart, liver, spleen, lung, kidney) at 14 and 28 days post-treatment showed no pathological lesions, suggesting the in vivo safety of DRDENs and naringenin chalcone (Fig. 5B).

Oral ingestion is the most common and noninvasive way of drug administration. To clarify DRDENs’ resistance to gastric acid digestion, we incubated DRDENs in a gastric acid simulation solution for 1 h at 37 °C. It was found that DRDENs remained intact in a highly acidic simulated gastric solution (Supplementary Figure S4a), although their concentration decreased by half (Supplementary Figure S4b).

After 14 and 28 days of oral administration of the DRDENs and naringenin chalcone, we adopted micro-CT scanning to detect bone regeneration in maxillary first molar (M1) extraction sockets (Fig. 5C). The analysis revealed that DRDENs and naringenin chalcone treatment significantly increased BV/TV and BMD of the extraction socket compared to the control group (Fig. 5D), indicating that DRDENs and naringenin chalcone may prevent bone loss and enhance bone mass in mice.

**Fig. 6:**
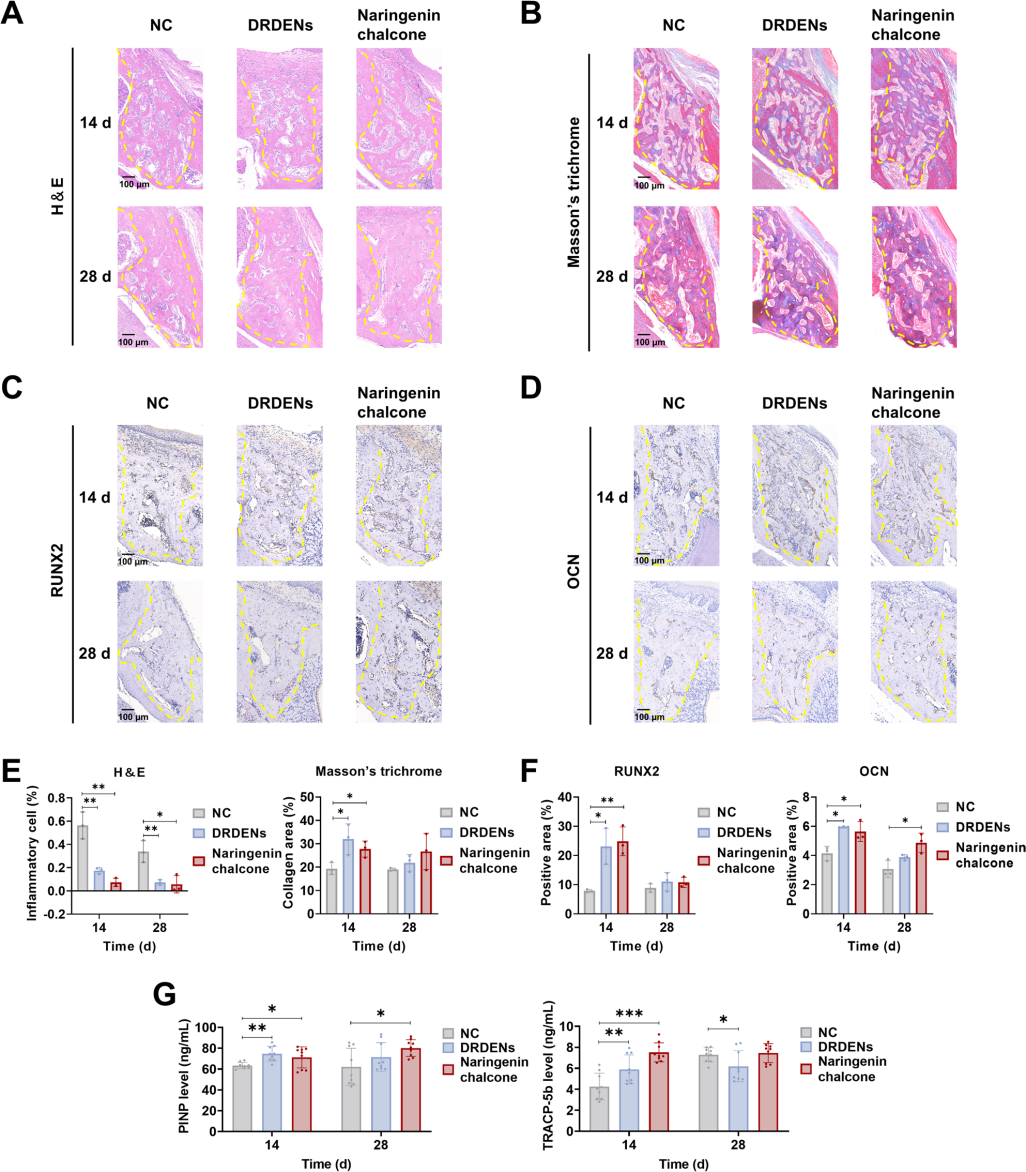
Histological staining and ELISA assay showing alveolar bone healing in tooth extraction mice with DRDENs and naringenin chalcone treated. (**A**) H&E staining in the M1 alveolar socket (Scale bar: 100 μm). (**B**) Masson’s trichrome staining in the M1 alveolar socket (Scale bar: 100 μm). (**C**-**D**) Immunohistochemistry staining of RUNX2 and OCN in the M1 alveolar socket (Scale bar: 100 μm). (**E**) Quantitative analysis of inflammatory cell infiltration area in H&E staining and collagen area of new bone in Masson’s trichrome staining. (**F**) Quantitative analysis of positive stained area of RUNX2 and OCN. (**G**) PINP and TRACP-5b levels in mice serum measured by ELISA

On the H&E and Masson’s trichrome staining analysis of maxillary M1 extraction sockets, less inflammatory cell infiltration and increased new bone formation were observed in the DRDENs and naringenin chalcone groups (Fig. 6A, B, E). Additionally, IHC staining confirmed upregulated expression of RUNX2 and OCN in the extraction socket region (Fig. 6C, D, F), consistent with enhanced osteogenic activity.

Bone reconstruction is a dynamic balance process between bone formation and resorption. To assess the general bone regeneration, mouse serum was collected at 14 and 28 days post-administration. We measured the bone-forming marker procollagen I N-terminal propeptide (PINP) and the bone resorptive marker tartrate-resistant acid phosphatase-5b (TRACP-5b) by ELISA. PINP is a peptide fragment produced in the synthesis of type I collagen. PINP levels can directly reflect bone formation activity. On the contrary, TRACP-5b is a specific marker secreted by osteoclasts. By measuring serum TRACP-5b concentration, bone resorptive activity would be estimated [34]. In the 14 days post-treatment, both PINP and TRACP-5b are significantly increased, indicating enhanced bone remodeling. At 28 days post-treatment, the naringenin chalcone group showed a significant increase in PINP, and the DRDENs group showed a remarkable decrease in TRACP-5b (Fig. 5G). suggesting sustained bone formation.

### Mechanism of DRDENs-mediated osteogenesis: naringenin chalcone-ITPR3-calcium flux axis

**Fig. 7:**
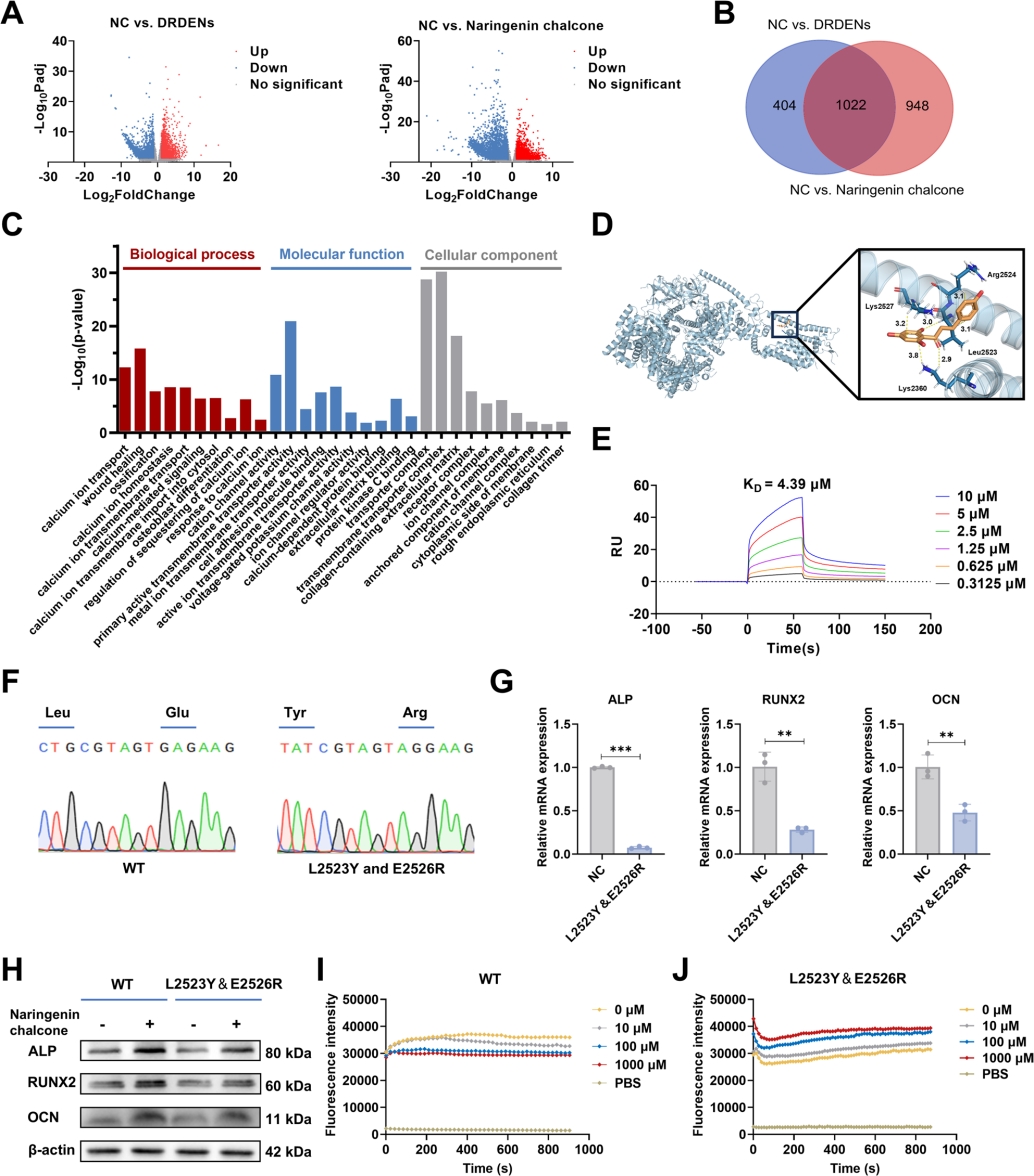
Activation of ITPR3 by naringenin chalcone released by DRDENs (**A**) Volcano plots of differentially expressed genes between the negative control group vs. DRDENs group and the negative control group vs. naringenin chalcone group, analyzed by transcriptome sequencing of the tooth extraction sockets at 14d post procedure. (**B**) Venn diagram showing overlapping genes between the negative control group vs. the DRDENs group and the negative control group vs. the naringenin chalcone group. (**C**) Gene Ontology (GO) enrichment analysis for genes in the purple module of the Venn plot. (**D**) 3D visualization analysis of molecular docking between ITPR3 and naringenin chalcone. (**E**) Interaction between ITPR3 and naringenin chalcone measured by SPR. (**F**) Sequencing validation of L2523Y and E2526R mutated hDFSCs constructed by the CRISPR-Cas9 technique. (**G**) ALP, RUNX2, and OCN mRNA expression levels in the WT hDFSCs group and the mutated hDFSCs group. (**H**) Western blot analysis in the WT hDFSCs group and the mutated hDFSCs group with and without naringenin chalcone treatment. (**I**-**J**) Calcium flux in the WT hDFSCs group and the mutated hDFSCs group treated with different concentrations of naringenin chalcone

To elucidate the mechanisms underlying DRDENs, transcriptomic sequencing was performed. Mice with maxillary M1 extraction treated with DRDENs and naringenin chalcone were euthanized at the time of the greatest bone repair response on day 14 [35]. The maxilla with the extraction sites was dissected and subjected to RNA-sequencing analysis. As shown in the volcano plots (Fig. 7A), many genes were differentially expressed between the two groups. According to a Venn diagram of two comparative gene lists (Fig. 7B) and Gene Ontology (GO) analysis (Fig. 7C), we observed that calcium ion-related biological processes and molecular functions are remarkably enriched. Since the endoplasmic reticulum is a central place for calcium storage and release, the endoplasmic reticulum-localized calcium channel receptor, inositol 1,4,5-trisphosphate receptor type 3 (ITPR3), was selected as a potential target.

Molecular docking is a preliminary screening tool for the interaction between ligand and receptor. Docking between naringenin chalcone and ITPR3 was performed. In thermodynamics, the lower the binding free energy, the more stable a complex is [36]. It is computed that the binding affinity of naringenin chalcone (-7.1 kcal/mol) was stronger than the endogenous ligand inositol triphosphate (IP3) (-5.4 kcal/mol) (Supplementary Figure S5a). The greater affinity of naringenin chalcone suggests it may be a competitive ligand for ITPR3. Subsequently, the binding conformation and interaction force of naringenin chalcone with ITPR3 were analyzed and visualized in 3D (Fig. 7D) and 2D plots (Supplementary Figure S5b).

Molecular dynamics simulation is a computational technique for investigating the dynamic interactions between the ligand-receptor complex. We conducted a molecular dynamics simulation of naringenin chalcone and ITPR3. In the results, RMSD is a parameter that reflects the complex’s movement. As shown in Supplementary Figure S6a, the protein simulation converged at about 45 ns and fluctuated stably within 2 Å. The early-stage convergence and low volatility imply that the binding action was close and steady. Additionally, RMSF is another parameter used to assess the protein receptor’s flexibility during simulation. There was an evident decline in the RMSF diagram at about 2500 amino acids in ITPR3, indicating that the complex had become stable at this position (Supplementary Figure S6b). The region around this may be the functional site where naringenin chalcone binds in ITPR3. RoG was also computed, providing information on the protein’s compactness in the simulation. The decrease in RoG of ITPR3 indicates that the structure was overall compact (Supplementary Figure S6c). Finally, we calculated the SASA, which exhibits the complex’s interactions with its surrounding solvent. As shown in Supplementary Figure S6d, the SASA declined from about 30 ns, indicating that the compound’s surface was well-embedded and may have contributed to effective integration between the ligand and receptor. The binding free energy of the system was computed using the MM/GBSA algorithm, yielding − 24.21 ± 2.59 kcal/mol. The other energy values were shown in Supplementary Table S3. In summary, the dynamic simulation results support the binding of naringenin chalcone and ITPR3.

To solidly demonstrate the interaction between naringenin chalcone and ITPR3, surface plasmon resonance (SPR) was used. SPR is an optical sensing technology that quantitatively and sensitively detects interactions between two molecules. Based on the results of the molecular dynamics simulation above, we selected the ITPR3 fragment (2472–2582 aa) to provide the receptor protein. As shown in Fig. 7E, the K_d_ value was 4.39 µM, providing strong evidence of strong interactions. The association rate constant and dissociation rate constant are provided in Supplementary Table S4.

Subsequently, the exact binding sites of ITPR3 were researched. We ranked the binding free energies of amino acid residues calculated in the MM/GBSA analysis (Supplementary Figure S6e, f) and selected those with binding energies below − 1 kcal/mol for computational alanine scanning. It was shown that the mutation energies of Leu2523 and Glu2526 were over 0.5 kcal/mol (Supplementary Figure S7a), suggesting that changes in these two amino acids would weaken the affinity between naringenin chalcone and ITPR3. Hence, we selected Leu2523 and Glu2526 for saturation mutagenesis. Further, in the saturation mutagenesis, it is predicted that the affinity between the naringenin chalcone and ITPR3 may substantially decrease when Leu2523 mutates into Tyr2523 and Glu2526 mutates into Arg2526 (Supplementary Figure S7b).

The CRISPR/Cas9 technique was then used to introduce point mutations at L2523Y and E2526R in ITPR3 in hDFSCs (Fig. 7F). Wild-type (WT) and mutated hDFSCs were cultured and treated with naringenin chalcone. After that, the cells were collected for qRT-PCR and western blot analysis. In qRT-PCR results (Fig. 7G), the osteogenic gene levels of ALP, RUNX2, and OCN were downregulated when the point mutations of L2523Y and E2526R in ITPR3 were introduced, indicating that the mutated sites may be critical for osteogenesis. Moreover, in western blotting (Fig. 7H), the osteogenic proteins ALP, RUNX2, and OCN were decreased, consistent with the gene-level results. Expression of these osteogenic markers was upregulated by naringenin chalcone treatment in both WT and point-mutated groups. However, their expressions were lower in the point-mutated group than in the WT group. These results demonstrated that naringenin chalcone could bind to ITPR3 and regulate osteogenic effects, and that Leu2523 and Glu2526 are critical active binding sites between naringenin chalcone and ITPR3.

Since ITPR3 is responsible for the calcium flow and storage, we measured the dynamic intracellular calcium using the endoplasmic reticulum-specific fluorescent dye Mag-fluo-4 AM. As shown in Fig. 7I, fluorescence intensities declined with the increase of naringenin chalcone in WT hDFSCs, indicating the release of calcium. It is shown that the calcium fluorescence signals in L2523Y and E2526R mutated hDFSCs were intensified with increasing naringenin chalcone (Fig. 7J). The opposite calcium flux response in WT and point-mutated hDFSCs further demonstrated that ITPR3 was a key target of naringenin chalcone.

Taken together, we demonstrated that DRDENs exert osteogenic effects on alveolar socket healing by endocytosis and/or naringenin chalcone release, which bind to ITPR3 and activate the calcium flux.

### Quantitative analysis of naringenin chalcone in DRDENs

In the UPLC-MS/MS analysis of DRDENs above, it was shown that naringenin chalcone appears to have the highest peak area. While peak area reflects the abundance of the molecule, we measured the level of naringenin chalcone in DRDENs. A standard curve of naringenin chalcone was established, and a regression equation was obtained (Supplementary Figure S8). Using the formula based on the standard curve and area calculations of naringenin chalcone in DRDENs, we found that the concentration of naringenin chalcone in DRDENs was about 0.3748 ± 0.2191 µM.

### Function identification of Drynaria roosii CHI

Production of naringenin chalcone in *Drynaria roosii* is influenced by biosynthetic enzymes inside. To identify approaches to regulate naringenin chalcone production, we explored chalcone isomerase (CHI) in *Drynaria roosii*. CHI is a key rate-limiting enzyme in the flavonoid biosynthesis pathway, and can be categorized into types I, II, III, and IV. Type I CHIs can only catalyze the stereospecific isomerization of naringenin chalcone, while type II CHIs can further catalyze isoliquiritigenin into liquiritigenin [37]. Although the cyclization reaction of naringenin chalcone can occur spontaneously, the catalytic efficiency of CHI was 10^7^ times higher [38]. Type III CHIs participate in fatty acid metabolism [39], while type IV CHIs appear to be rectifiers in plant flavonoid biosynthesis. Both type III and type IV CHIs have no enzymatic activity [40]. The CHI function in *Drynaria roosii* remained unclear.

**Fig. 8:**
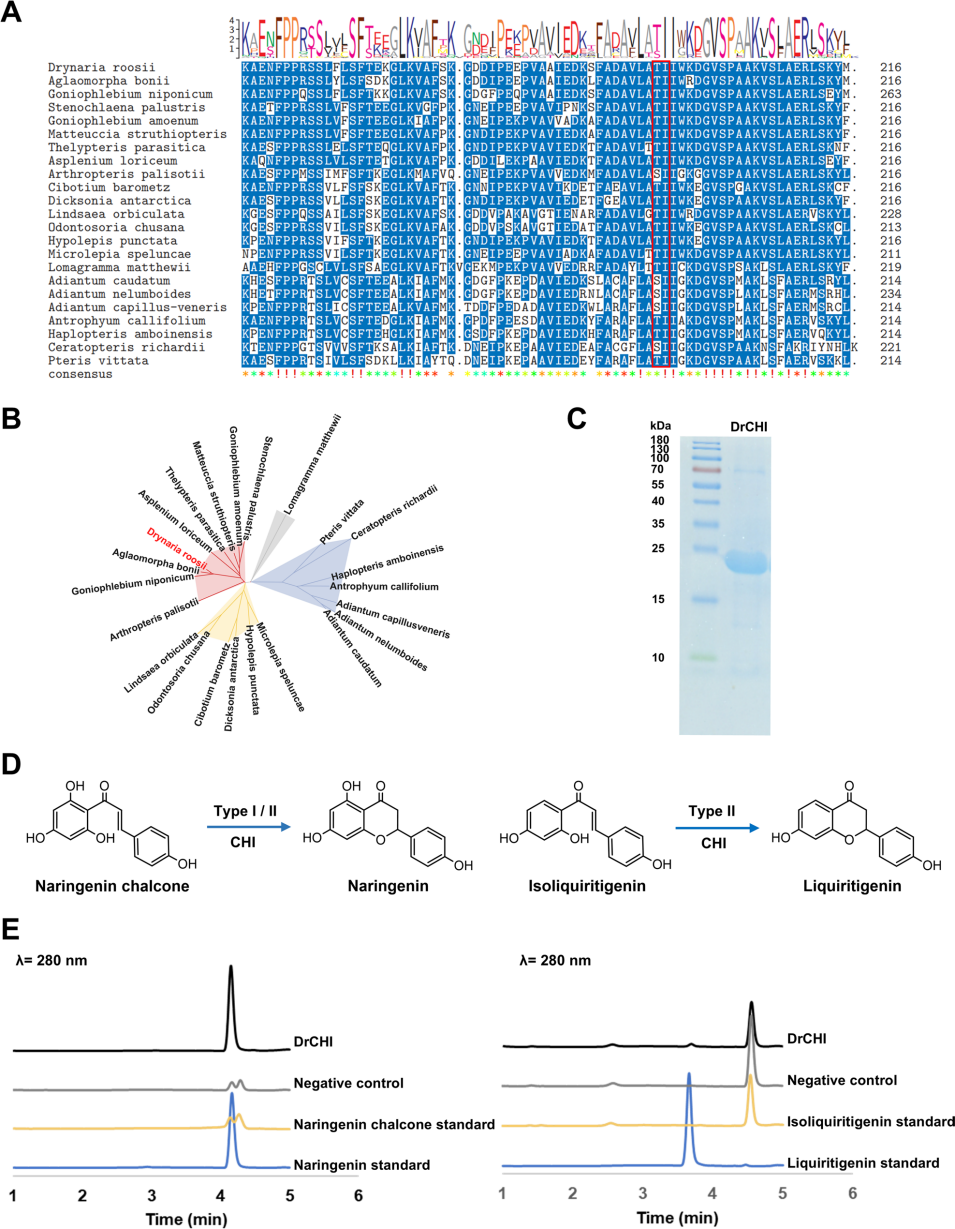
Identification of chalcone isomerase in Drynaria roosii. (**A**) Multiple sequence alignment of the CHI in *Drynaria roosii* with 22 other pteridophyte species (Blue shade indicates identical residues; red box indicates residues that affect substrate preference; dot indicates a gap). (**B**) Phylogenetic analysis of the DrCHI with other pteridophytes. (**C**) SDS-PAGE analysis of the purified DrCHI. (**D**) Illustration of the reactions catalysed by type I CHI and type II CHI. (**E**) LC/MS analysis of the enzyme reactions of DrCHI with naringenin chalcone, and isoliquiritigenin

A publicly available transcriptomic database of *Drynaria roosii* on NCBI was then found [41]. We downloaded the RNA sequence of *Drynaria roosii* to establish a local database and translate it into amino acid sequences. Several known chalcone isomerase (CHI) sequence from ferns was used as probes to match and splice the CHI sequence of *Drynaria roosii* (DrCHI).

To confirm the sequence of DrCHI, we extracted total RNA from *Drynaria roosii* and reverse-transcribed it into cDNA. With the cDNA template, *DrCHI* DNA was amplified and gene-sequenced (Supplementary Figure S9a and Table S5). In the genetic sequencing result, we noticed several bases (CTC, AAA, and CCT) differed from those in the database (CTT, AAG, and CCA). By referring to codon-degeneracy data, we found that the genetic codes mapped to the same amino acids (Leu, Lys, and Pro) (Supplementary Figure S9b).

We then used the Basic Local Alignment Search Tool (BLAST) to analyze the DrCHI sequence. 22 ferns with 60.58%-96.30% identity were matched (Supplementary Table S6). These sequences were further used for multiple sequence alignment analysis and phylogenetic tree construction. In multiple sequence alignment analysis, the residues postulated to determine the substrate preference of CHIs [42] were indicated by a red box (Fig. 8A). And in the phylogenetic tree, it was shown that *Aglaomorpha bonii* is most closely related to *Drynaria roosii* (Fig. 8B).

Recently, a study on CHIs in ferns reports that five ferns (*Pteris vittata*, *Haplopteris amboinensis*, *Antrophyum callifolium*, *Adiantum capillus*, *Adiantum caudatum*) with Ser193/Ile194 or Thr193/Ile194 exhibited weak type II CHI activity (using the CHI numbering scheme from *Drynaria roosii* in the current study). The researchers found that all five ferns have a conservative residue at Phe190, whereas the corresponding position in other type I CHIs is Val190. They mutated Phe190 to Val190 in the five CHIs and found that all five CHIs turned into complete type I CHIs [43]. In our study, the DrCHI in alignment sequences was Thr193/Ile194 and Val190 (Fig. 8A). Thus, DrCHI may be predicted as a complete type I CHI.

The *DrCHI* fragment was then ligated into the pET28a plasmid vector. The recombinant plasmid harboring the DrCHI fragment was transferred into *E. coli* DH5α and *E. coli* Rosetta. Upon IPTG induction, the DrCHI protein was expressed and purified (Fig. 8C). Next, we used naringenin chalcone and isoliquiritigenin as substrates, respectively, and detected the enzymatic reaction products by LC-MS. The results showed that DrCHI efficiently catalyzed naringenin chalcone and catalyzed isoliquiritigenin to liquiritigenin only slightly, revealing its type I CHI function with weak type II CHI activity (Fig. 8D, E). The function of DrCHI was subtly different from the prediction above. We speculated that the reason was that the previous law was summarized from five ferns on the same clade, while *Drynaria roosii* was in a relatively distant clade (Fig. 8B). Future studies on the knockout or editing of *DrCHI* may contribute to the accumulation and production of naringenin chalcone in *Drynaria roosii* and DRDENs.

## Discussion

Alveolar bone loss after tooth extraction remains a major clinical challenge, highlighting the need for effective bone regeneration strategies. Plant-derived exosome-like nanovesicles have emerged as promising therapeutic agents due to their natural origin, biocompatibility, and drug delivery capabilities [44]. In this study, we isolated and characterized DRDENs from *Drynaria roosii*, a medicinal plant with known bone-protective effects [45], and identified naringenin chalcone as a novel bioactive component of *Drynaria roosii*. Our findings demonstrate that DRDENs and naringenin chalcone promote alveolar socket healing via ITPR3-mediated calcium flux, providing a new therapeutic approach for post-extraction bone loss. DRDENs exhibited typical exosome-like characteristics, with a spherical morphology, uniform size distribution, and negative ζ-potentials. The consistent HPLC fingerprint of seven batches confirms the reproducibility of the isolation method, which is critical for translational research. Cellular uptake studies showed that DRDENs are efficiently internalized by osteoblasts and stem cells, enabling the delivery of bioactive cargo. In vitro biocompatibility assays (i.e., cytotoxicity and hemolysis) and i*n vivo* histological analysis of major organs confirmed the safety of DRDENs and naringenin chalcone, supporting their potential clinical application. Before clinical translation, several questions, such as large-scale preparation, batch-to-batch consistency, and detailed quality control guidance for DRDENs, needed to be addressed and established.

Regarding the absorption, systemic circulation, and tissue accumulation of plant-derived nanovesicles in oral delivery, it may be inferred that after oral administration, the nanovesicles pass through the stomach and then reach the intestine. From the intestine, nanovesicles penetrate the blood capillary endothelium to enter the bloodstream [46]. In inflamed or injured bone tissues (e.g., alveolar socket vessels), vascular permeability increases, allowing nanovesicles to extravasate and accumulate.

About 7.21% oral bioavailability was reported in grape-derived nanovesicles and 4.87% oral bioavailability in ginger-derived nanovesicles [47]. The bioavailability of oral administration is usually lower than that of intravenous or intraperitoneal administration, but it is noninvasive, painless, and safe.

It is documented that the tissue distribution of tea leaf-derived nanovesicles by oral delivery could be located in the liver, spleen, lung, heart, kidney, and gastrointestinal tract in 48 h [48]; however, no signal was detected in the same organs of the *Catharanthus roseus*-derived nanovesicles orally administered group within 48 h, except for the gastrointestinal tract [49]. In our study, after administering the drug orally, we did not conduct a time-series dissection. Instead, we dissected and imaged the major organs of the mice after continuous monitoring in vivo for 48 h. DRDENs were mainly distributed in the abdominal region in vivo after oral administration 24 h earlier. 48 h later, ex vivo imaging showed barely any fluorescence signals in the oral delivery group compared to the intraperitoneal and tail vein injection groups. While the cranio-maxillary bone had a slightly lower fluorescence signal than the intraperitoneal and tail vein injection groups, indicating that the oral route has lower bioavailability and faster drug evacuation capacity, with the advantage of being less burdensome to organs, and the ability to exert biological effects on the cranio-maxillary bone.

Naringenin chalcone was identified as the dominant bioactive component in DRDENs, with concentration-dependent osteogenic activity in MC3T3-E1 cells and hDFSCs. Stability studies revealed that naringenin chalcone is unstable in PBS but stable in normal saline, with pH-dependent degradation. The interaction between naringenin chalcone and BSA suggests potential serum stability, which is essential for in vivo applications. DRDENs may act as protective carriers for naringenin chalcone, enhancing its stability and bioavailability.

Mechanistically, we uncovered a novel pathway by which naringenin chalcone binds to ITPR3, activating ER calcium release and promoting osteogenic differentiation. ITPR3 is a key regulator of calcium homeostasis, and its activation has been linked to bone formation [50]. Our results show that naringenin chalcone binds ITPR3 with higher affinity than its endogenous ligand, IP3, suggesting competitive activation. Critical binding residues (Leu2523 and Glu2526) were identified, and point mutations in these residues weakened naringenin chalcone’s osteogenic effect, confirming ITPR3 as a direct target. Calcium flux measurements further validated that naringenin chalcone induces ITPR3-mediated ER calcium release, which is known to regulate osteoblast proliferation and differentiation [51].

Additionally, we identified DrCHI as a type I CHI with weak type II CHI activity, mainly catalyzing the conversion of naringenin chalcone. Previous studies reported that fern CHIs with Ser193/Ile194 or Thr193/Ile194 and Phe190 exhibit weak type II activity [43], but DrCHI contains Val190 and still functions as a weak type II CHI. These suggest that the classification of CHI subtypes is more complex than previously thought, possibly influenced by phylogenetic background. Although the CHI from *Drynaria roosii* has a weak type II CHI activity, which is much lower than that of the typical type II CHI, the classification of the CHI folding protein family is based on substrate specificity, phylogenetic relationship, sequence homology, etc., rather than the strength of activity. Therefore, the CHI of *Drynaria roosii* remains of type I, and its weak CHI activity may be an evolutionary remnant. Identification of DrCHI provides a basis for genetic engineering to enhance naringenin chalcone production in *Drynaria roosii*.

This study has several limitations. First, the in vivo experiments were performed in mice, and translation to humans requires further validation in large-animal models and clinical trials. Second, Other potential bioactive components in DRDENs may contribute to bone healing, and their synergistic effects with naringenin chalcone warrant further investigation. From the highest to the lowest in terms of abundance, other components include eriodictyol, naringin, narirutin, eriocitrin, procyanidin B2, aloin, engeletin, betaine, astilbin, and α-cyperone. All of them had been reported to have therapeutic effects in bone disorders [52–61], and they may contribute to DRDENs based on their content weight. Third, the osteoclast-related responses of DRDENs and naringenin chalcone need to be evaluated in the future. Four, local delivery to the extraction socket may also be an effective and more direct drug administration way worth investigating. Compared with local treatment, oral administration used in the current study is relatively indirect, but it can still reach the target site via systemic absorption and exert its efficacy locally within the alveolar socket after the wound surface closes. In the meantime, systemic absorption after oral administration may improve the overall microenvironment, thereby benefiting local healing.

In conclusion, our study demonstrates that DRDENs promote alveolar socket healing via naringenin chalcone-mediated ITPR3 activation and calcium flux regulation. We also identified DrCHI as a type I CHI with weak type II CHI activity involved in naringenin chalcone biosynthesis. These findings highlight the therapeutic potential of DRDENs and naringenin chalcone for bone regeneration and provide new insights into the molecular mechanisms of plant-derived nanovesicles in tissue repair.

## Electronic Supplementary Material

Below is the link to the electronic supplementary material.

Supplementary Material 1.

Supplementary Material 2.

## Data Availability

Data supporting the findings of this work are available within the paper and its Supplementary Information files. Additional data related to this paper are available from the corresponding author on reasonable request.

## References

[1] Udeabor SE, Heselich A, Al-Maawi S, Alqahtani AF, Sader R, Ghanaati S. Current knowledge on the healing of the extraction socket: A narrative review. Bioengineering. 2023;10(10):1145. 37892875 10.3390/bioengineering10101145 PMC10604628

[2] Geng YA-O, Bao C, Chen Y, Yan Z, Miao F, Wang T, Li Y, Li L, Sun W, Xu Y. NLRP3 deficiency improves bone healing of tooth extraction sockets through SMAD2/3-RUNX2-mediated osteoblast differentiation. Stem Cells. 2024;42(12):1085–99. 39404121 10.1093/stmcls/sxae064

[3] Evian CI, Rosenberg ES, Coslet JG, Corn H. The osteogenic activity of bone removed from healing extraction sockets in humans. J Periodontol‌. 1982;53(2):81–5. 6950085 10.1902/jop.1982.53.2.81

[4] Tan WL, Wong TLT, Wong MCM, Lang NP. A systematic review of post-extractional alveolar hard and soft tissue dimensional changes in humans. Clin Oral Implants Res. 2011;23(s5):1–21. 22211303 10.1111/j.1600-0501.2011.02375.x

[5] Akers JC, Gonda D, Kim R, Carter BS, Chen CC. Biogenesis of extracellular vesicles (EV): exosomes, microvesicles, retrovirus-like vesicles, and apoptotic bodies. J Neuro-Oncol‌. 2013;113(1):1–11. 10.1007/s11060-013-1084-8 PMC5533094 23456661

[6] Jeppesen DK, Zhang Q, Franklin JL, Coffey RJ. Extracellular vesicles and nanoparticles: emerging complexities. Trends Cell Biol‌. 2023;33(8):667–81. 36737375 10.1016/j.tcb.2023.01.002 PMC10363204

[7] Nagelkerke A, Ojansivu M, van der Koog L, Whittaker TE, Cunnane EM, Silva AM, Dekker N, Stevens MM. Extracellular vesicles for tissue repair and regeneration: evidence, challenges and opportunities. Adv Drug Deliv Rev. 2021;175:113775. 33872693 10.1016/j.addr.2021.04.013

[8] Hiltbrunner S, Larssen P, Eldh M, Martinez-Bravo MJ, Wagner AK, Karlsson MC, Gabrielsson S. Exosomal cancer immunotherapy is independent of MHC molecules on exosomes. Oncotarget. 2016;7(25):38707–17. 27231849 10.18632/oncotarget.9585 PMC5122422

[9] Meng W, He C, Hao Y, Wang L, Li L, Zhu G. Prospects and challenges of extracellular vesicle-based drug delivery system: considering cell source. Drug Deliv‌. 2020;27(1):585–98. 32264719 10.1080/10717544.2020.1748758 PMC7178886

[10] You JY, Kang SJ, Rhee WJ. Isolation of cabbage exosome-like nanovesicles and investigation of their biological activities in human cells. Bioactive Mater. 2021;6(12):4321–32. 10.1016/j.bioactmat.2021.04.023 PMC8105599 33997509

[11] Lei C, Mu J, Teng Y, He L, Xu F, Zhang X, Sundaram K, Kumar A, Sriwastva MK, Lawrenz MB, et al. Lemon exosome-like nanoparticles-manipulated probiotics protect mice from C. diff infection. iScience. 2020;23(10):101571. 33083738 10.1016/j.isci.2020.101571 PMC7530291

[12] Gao C, Zhou Y, Chen Z, Li H, Xiao Y, Hao W, Zhu Y, Vong CT, Farag MA, Wang Y, et al. Turmeric-derived nanovesicles as novel nanobiologics for targeted therapy of ulcerative colitis. Theranostics. 2022;12(12):5596–614. 35910802 10.7150/thno.73650 PMC9330521

[13] Hwang J-H, Park Y-S, Kim H-S, Kim D-h, Lee S-H, Lee C-H, Lee S-H, Kim J-E, Lee S, Kim HM, et al. Yam-derived exosome-like nanovesicles stimulate osteoblast formation and prevent osteoporosis in mice. J Control Release. 2023;355:184–98. 36736431 10.1016/j.jconrel.2023.01.071

[14] Zhang W, Song Q, Bi X, Cui W, Fang C, Gao J, Li J, Wang X, Qu K, Qin X, et al. Preparation of Pueraria lobata root-derived exosome-like nanovesicles and evaluation of their effects on mitigating alcoholic intoxication and promoting alcohol metabolism in mice. Int J Nanomed. 2024;19:4907–21. 10.2147/IJN.S462602 PMC11141763 38828197

[15] Dvorakova M, Soudek P, Pavicic A, Langhansova L. The traditional utilization, biological activity and chemical composition of edible fern species. J Ethnopharmacol. 2024;324:117818. 38296173 10.1016/j.jep.2024.117818

[16] Jin H, Jiang NN, Xu WS, Zhang ZY, Yang Y, Zhang JM, Xu H. Effect of flavonoids from Rhizoma drynariae on osteoporosis rats and osteocytes. Biomed Pharmacother‌. 2022;153:113379. 36076521 10.1016/j.biopha.2022.113379

[17] Xu Z-L, Xu M-Y, Wang H-T, Xu Q-X, Liu M-Y, Jia C-P, Geng F, Zhang N. Pharmacokinetics of eight flavonoids in rats assayed by UPLC-MS/MS after oral administration of Drynariae rhizoma extract. J Anal Methods Chem. 2018;2018:1–11. 10.1155/2018/4789196 PMC6312611 30662789

[18] Tian Y, Bai D, Guo W, Li J, Zeng J, Yang L, Jiang Z, Feng L, Yu M, Tian W. Comparison of human dental follicle cells and human periodontal ligament cells for dentin tissue regeneration. Regen Med. 2015;10(4):461–79. 26022765 10.2217/rme.15.21

[19] Zhang HJ, Li ZX, Zhou S, Li SM, Ran HM, Song ZL, Yu T, Yin WB. A fungal NRPS-PKS enzyme catalyses the formation of the flavonoid naringenin. Nat Commun. 2022;13(1):6361. 36289208 10.1038/s41467-022-34150-7 PMC9606254

[20] Zhao Q, Feng J, Liu F, Liang Q, Xie M, Dong J, Zou Y, Ye J, Liu G, Cao Y, et al. Rhizoma drynariae-derived nanovesicles reverse osteoporosis by potentiating osteogenic differentiation of human bone marrow mesenchymal stem cells via targeting ERα signaling. Acta Pharm Sinica B. 2024;14(5):2210–27. 10.1016/j.apsb.2024.02.005 PMC11119514 38799625

[21] Yoshimura M, Sano A, Kamei JI, Obata A. Identification and quantification of metabolites of orally administered naringenin chalcone in rats. J Agric Food Chem. 2009;57(14):6432–7. 19558184 10.1021/jf901137x

[22] Zhuang X, Deng ZB, Mu J, Zhang L, Yan J, Miller D, Feng W, McClain CJ, Zhang HG. Ginger-derived nanoparticles protect against alcohol-induced liver damage. J Extracell Vesicles. 2015;25(4):28713. 10.3402/jev.v4.28713 PMC4662062 26610593

[23] Eberhardt J, Santos-Martins D, Tillack AF, Forli S. AutoDock Vina 1.2.0: New docking methods, expanded force field, and python bindings. J Chem Inf Model‌. 2021;61(8):3891–8. 34278794 10.1021/acs.jcim.1c00203 PMC10683950

[24] Case DA, Aktulga HM, Belfon K, Cerutti DS, Cisneros GA, Cruzeiro VWD, Forouzesh N, Giese TJ, Götz AW, Gohlke H, et al. : AmberTools. J Chem Inf Model‌. 2023;63(20):6183–91. 37805934 10.1021/acs.jcim.3c01153 PMC10598796

[25] Pattnaik P. Surface plasmon resonance: applications in understanding receptor-ligand interaction. Appl Biochem Biotechnol. 2005;126(2):79–92. 16118464 10.1385/abab:126:2:079

[26] Delgado J, Radusky LG, Cianferoni D, Serrano L, Valencia A. FoldX 5.0: working with RNA, small molecules and a new graphical interface. Bioinformatics. 2019;35(20):4168–9. 30874800 10.1093/bioinformatics/btz184 PMC6792092

[27] Rossi AM, Taylor CW. Reliable measurement of free Ca2 + concentrations in the ER lumen using Mag-Fluo-4. Cell Calcium. 2020;87:102188. 32179239 10.1016/j.ceca.2020.102188 PMC7181174

[28] Letunic I, Bork P. Interactive Tree of Life (iTOL) v6: recent updates to the phylogenetic tree display and annotation tool. Nucleic Acids Res. 2024;52(W1):W78–82. 38613393 10.1093/nar/gkae268 PMC11223838

[29] Tatsumi N, Tsuda I, Masaoka M, Imai K. Measurement of the zeta potential of human platelets by the use of laser-light scattering. Thromb Res. 1992;65(4–5):585–92. 1615497 10.1016/0049-3848(92)90208-r

[30] Zhou X, Li Z, Sun W, Yang G, Xing C, Yuan L. Delivery efficacy differences of intravenous and intraperitoneal injection of exosomes: perspectives from tracking dye labeled and miRNA encapsulated exosomes. Curr Drug Deliv‌. 2020;17(3):186–94. 31969102 10.2174/1567201817666200122163251

[31] Zhu Y, Yang Y, Lan Y, Yang Z, Gao X, Zhou J. The role of PKM2-mediated metabolic reprogramming in the osteogenic differentiation of BMSCs under diabetic periodontitis conditions. Stem Cell Res Ther. 2025;16(1):186. 40251642 10.1186/s13287-025-04301-w PMC12008901

[32] Martins CM, de Azevedo Queiroz IO, Ervolino E, Cintra LTA, Gomes-Filho JE. RUNX‐2, OPN and OCN expression induced by grey and white mineral trioxide aggregate in normal and hypertensive rats. Int Endod J. 2017;51(6):641–8. 29143348 10.1111/iej.12876

[33] Mol JNM, Robbinst MP, Dixon RA, Veltkamp E. Spontaneous and enzymic rearrangement of naringenin chalcone to flavanone. Phytochemistry. 1985;24(10):2267–9.

[34] Sølling AS, Harsløf T, Jørgensen NR, Langdahl B. Changes in RANKL and TRAcP 5b after discontinuation of denosumab suggest RANKL mediated formation of osteoclasts results in the increased bone resorption. Osteoporos Int‌. 2022;34(3):599–605. 36543965 10.1007/s00198-022-06651-0

[35] de Oliveira Puttini I, Gomes-Ferreira PHS, de Oliveira D, Hassumi JS, Gonçalves PZ, Okamoto R. Teriparatide improves alveolar bone modelling after tooth extraction in orchiectomized rats. Arch Oral Biol. 2019;102:147–54. 31022626 10.1016/j.archoralbio.2019.04.007

[36] Ren LCC, Wang RN, Wang Y, Tie FF, Dong Q, Wang HL, Hu N. Exploring the effect and mechanism of Hippophae rhamnoides L. triterpenoid acids on improving NAFLD based on network pharmacology and experimental validation in vivo and in vitro. J Ethnopharmacol. 2024;335:118657. 39127115 10.1016/j.jep.2024.118657

[37] Dastmalchi M, Dhaubhadel S. Soybean chalcone isomerase: evolution of the fold, and the differential expression and localization of the gene family. Planta. 2014;241(2):507–23. 25385351 10.1007/s00425-014-2200-5

[38] Wang JY, Jiang YF, Sun T, Zhang CH, Liu XH, Li YS. Genome-wide classification and evolutionary analysis reveal diverged patterns of chalcone isomerase in plants. Biomolecules. 2022;12(7):961. 35883518 10.3390/biom12070961 PMC9313115

[39] Ngaki MN, Louie GV, Philippe RN, Manning G, Pojer F, Bowman ME, Li L, Larsen E, Wurtele ES, Noel JP. Evolution of the chalcone-isomerase fold from fatty-acid binding to stereospecific catalysis. Nature. 2012;485(7399):530–3. 22622584 10.1038/nature11009 PMC3880581

[40] Luo KY, Wang SP, Yang L, Luo S-l, Cheng J, Dong Y, Ning Y. Wang W-b: Evolutionary landscape of plant chalcone isomerase-fold gene families. Front Plant Sci. 2025;16:1559547. 40225028 10.3389/fpls.2025.1559547 PMC11985768

[41] Sun M-Y, Li J-Y, Li D, Huang F-J, Wang D, Li H, Xing Q, Zhu H-B, Shi L. Full-length transcriptome sequencing and modular organization analysis of the naringin/neoeriocitrin-related gene expression pattern in Drynaria roosii. Plant Cell Physiol. 2018;57(7):1398–414. 10.1093/pcp/pcy072 29660070

[42] Yu S, Li J, Peng T, Ni S, Feng Y, Wang Q, Wang M, Chu X, Fan Z, Li X, et al. Identification of chalcone isomerase family genes and roles of CnCHI4 in flavonoid metabolism in Camellia nitidissima. Biomolecules. 2022;13(1):41. 36671426 10.3390/biom13010041 PMC9855375

[43] Ni R, Zhu TT, Zhang XS, Wang PY, Sun CJ, Qiao YN, Lou HX, Cheng AX, Griffiths H. Identification and evolutionary analysis of chalcone isomerase-fold proteins in ferns. J Exp Bot. 2020;71(1):290–304. 31557291 10.1093/jxb/erz425 PMC6913697

[44] Mu N, Li J, Zeng L, You J, Li R, Qin A, Liu X, Yan F, Zhou Z. Plant-derived exosome-like nanovesicles: current progress and prospects. Int J Nanomed. 2023;18:4987–5009. 10.2147/IJN.S420748 PMC10492547 37693885

[45] Jang S-A, Hwang Y-H, Kim T, Lee A, Ha H. Anti-osteoporotic and anti-adipogenic effects of the water extract of Drynaria roosii nakaike in ovariectomized mice fed a high-fat diet. Molecules. 2019;24(17):3051. 31443447 10.3390/molecules24173051 PMC6749363

[46] Fang Z, Liu K. Plant-derived extracellular vesicles as oral drug delivery carriers. J Controll Release. 2022;350:389–400. 10.1016/j.jconrel.2022.08.046 36037973

[47] Zheng Y, Qin Y, He Q, Xie J, Lv X, Xiang T, Lan Z, Luo S, Liu Y, Xu M, et al. Enhanced delivery of oral biomacromolecules through edible plant-derived nanovehicles: exploiting the self-amplifying trancytosis feedback loop and phosphatidic acid. ACS Nano. 2025;20(1):1710–31. 41469236 10.1021/acsnano.5c20533

[48] Chen Q, Zu M, Gong H, Ma Y, Sun J, Ran S, Shi X, Zhang J, Xiao B. Tea leaf-derived exosome-like nanotherapeutics retard breast tumor growth by pro-apoptosis and microbiota modulation. J Nanobiotechnol. 2023;21(1):6. 10.1186/s12951-022-01755-5 PMC9811040 36600299

[49] Ou XZ, Wang HR, Tie HL, Liao JP, Luo YY, Huang WJ, Yu RM, Song LY, Zhu JH. Novel plant-derived exosome-like nanovesicles from Catharanthus roseus: preparation, characterization, and immunostimulatory effect via TNF-α/NF-κB/PU.1 axis. J Nanobiotechnol. 2023;21(1):160. 10.1186/s12951-023-01919-x PMC10199296 37210530

[50] Molitor A, Lederle A, Radosavljevic M, Sapuru V, Zavorka Thomas M, Yang J, Shirin M, Collin-Bund V, Jerabkova-Roda K, Miao Z, et al. A pleiotropic recurrent dominant ITPR3 variant causes a complex multisystemic disease. Sci Adv. 2024;10(37):aedi5545. 10.1126/sciadv.ado5545 PMC11397499 39270020

[51] Zayzafoon M. Calcium/calmodulin signaling controls osteoblast growth and differentiation. J Cell Biochem. 2006;97(1):56–70. 16229015 10.1002/jcb.20675

[52] Song F, Zhou L, Zhao J, Liu Q, Yang M, Tan R, Xu J, Zhang G, Quinn JMW, Tickner J, et al. Eriodictyol inhibits RANKL-induced osteoclast formation and function via inhibition of NFATc1 activity. J Cell Physiol. 2016;231(9):1983–93. 26754483 10.1002/jcp.25304

[53] Li Z, Gao K, Wang M, Liang S, Li D, Zhang P, Cheng W, Xu Z, Li N. Naringin inhibits the osteoblast-osteoclast pyroptosis cascade reaction mediated by accumulated bone marrow adipose tissue in the treatment of postmenopausal osteoporosis. J Orthop Transl. 2025;55:323–38. 10.1016/j.jot.2025.09.004 PMC12522718 41104349

[54] Wang Q, Peng X, Xu H, Zhao Y, Lu X, Lu C, Wang Q, Lu W, Sheng Q, Lu X, et al. Narirutin mitigates inflammatory arthritis and osteoporosis through modulating macrophage phenotype and osteoclastogenesis. J Orthop Transl. 2025;54:115–30. 10.1016/j.jot.2025.07.008 PMC12356004 40822517

[55] Yang J, Zheng W, Mei Y, Ni H, Liu H, Zhang Y, Li Y, Chen H, Cai L, Tian N, et al. Eriocitrin mitigates osteoarthritis development by suppressing chondrocyte inflammation and ECM degradation via Nrf2/HO-1/NF-κB pathways. J Agric Food Chem. 2025;73(49):31412–8. 41273287 10.1021/acs.jafc.5c11533

[56] Yamamoto T, Yuan H, Suzuki S, Nemoto E, Saito M, Yamada S. Procyanidin B2 enhances anti-inflammatory responses of periodontal ligament cells by inhibiting the dominant negative pro-inflammatory isoforms of peroxisome proliferator-activated receptor γ. J Dent Sci. 2024;19(3):1801–10. 39035263 10.1016/j.jds.2023.09.027 PMC11259626

[57] Li P, Kong J, Chen Z, Huang S, Lv G, Wei B, Wei J, Jing K, Quan J, Chu J. Aloin promotes osteogenesis of bone-marrow-derived mesenchymal stem cells via the ERK1/2-dependent Runx2 signaling pathway. J Nat Med. 2018;73(1):104–13. 30218208 10.1007/s11418-018-1249-z

[58] Feng M, Liu L, Wang J, Zhang J, Qu Z, Wang Y, He B. The molecular mechanisms study of engeletin suppresses RANKL-induced osteoclastogenesis and inhibits ovariectomized murine model bone loss. J Inflamm Res. 2023;16:2255–70. 37250105 10.2147/JIR.S401519 PMC10225148

[59] Wang M, Zhu J, Zheng X, Wu Z, Gao R. Betaine promotes the osteogenic differentiation of SCAPs via inhibiting NUMB. Tissue Cell. 2026;98:103142. 40974710 10.1016/j.tice.2025.103142

[60] Jin H, Wang Q, Chen K, Xu K, Pan H, Chu F, Ye Z, Wang Z, Tickner J, Qiu H, et al. Astilbin prevents bone loss in ovariectomized mice through the inhibition of RANKL-induced osteoclastogenesis. J Cell Mol Med. 2019;23(12):8355–68. 31603626 10.1111/jcmm.14713 PMC6850941

[61] Zhang H, Li S, Lu J, Jin J, Zhu G, Wang L, Yan Y, He L, Wang B, Wang X, et al. α-Cyperone (CYP) down-regulates NF-κB and MAPKs signaling, attenuating inflammation and extracellular matrix degradation in chondrocytes, to ameliorate osteoarthritis in mice. Aging. 2021;13(13):17690–706. 34237707 10.18632/aging.203259 PMC8312409

