# Supplementary material for: Drynaria roosii-derived exosome-like nanovesicles promote alveolar socket healing via activation of ITPR3-mediated calcium flux
Source: J Nanobiotechnology. 2026 May 20;24:659. doi: 10.1186/s12951-026-04530-y (PMC13366766; doi:10.1186/s12951-026-04530-y)
Supplement: Supplementary file 1 — Supplementary Material 1. [file 12951_2026_4530_MOESM1_ESM.docx]

**Supplementary information**

***Drynaria roosii*-derived exosome-like nanovesicles promote tooth extraction socket healing via activation of ITPR3-mediated calcium flux**

Yueting Lin^1^, Jiang Tao^1^*

1 Department of General Dentistry, Shanghai Ninth People’s Hospital, Shanghai Jiao Tong University School of Medicine; College of Stomatology, Shanghai Jiao Tong University; National Center for Stomatology; National Clinical Research Center for Oral Diseases; Shanghai Key Laboratory of Stomatology; Shanghai Research Institute of Stomatology. China

**Corresponding author:**

*Jiang Tao, taojiang_doctor@sjtu.edu.cn

Shanghai Ninth People’s Hospital, Shanghai Jiao Tong University School of Medicine, No. 500 Qu Xi Road, Shanghai, 200011, China.

**Contents**

**1 Supplementary Figures**

**Figure** **S1.** STR identification of MC3T3-E1 cells……………...………………………….3

**Figure S2.** Identification of hDFSCs……………………………………………………….4

**Figure S3.** Organs’ distribution of DRDENs in C57BL/6 after caudal vein injection, intraperitoneal injection, and oral administration……………………………………........4

**Figure S4.** In vitro digestion assay of DRDENs in simulated gastric solutions…………5

**Figure S5.** Molecular docking………………………………………………………………5

**Figure S6.** Molecular dynamics simulation…………………………………...…………...6

**Figure S7.** Virtual mutation of amino acid residues………………………...….…………7

**Figure S8.** Standard curve establishment of naringenin chalcone using LC-MS….….7

**Figure S9.** DNA fragment of *DrCHI*.……….………………...…….………………………7

**2 Supplementary Tables**

**Table S1.** Primers for qRT-PCR………………………….…………………………….......8

**Table S2.** Primers for DNA amplification and verification………………………....……...8

**Table S3.** Binding free energy calculated by MM/GBSA.…………………….………......8

**Table S4.** SPR result of ITPR3 and naringenin chalcone……………….…….………...9

**Table S5.** DNA and amino acid sequences of DrCHI…………………………….……....9

**Table S6.** Genbank accession number and sequences of 22 ferms CHIs used in multiple sequence alignment and phylogenetic analysis……………………….………...9

**Table S7.** Plasmids, strains, and cells used in this study………...…………………….12

**1 Supplementary Figures**

**Figure S1.** STR identification of MC3T3-E1 cells. a, The 1st passage. b, The 18th passage.


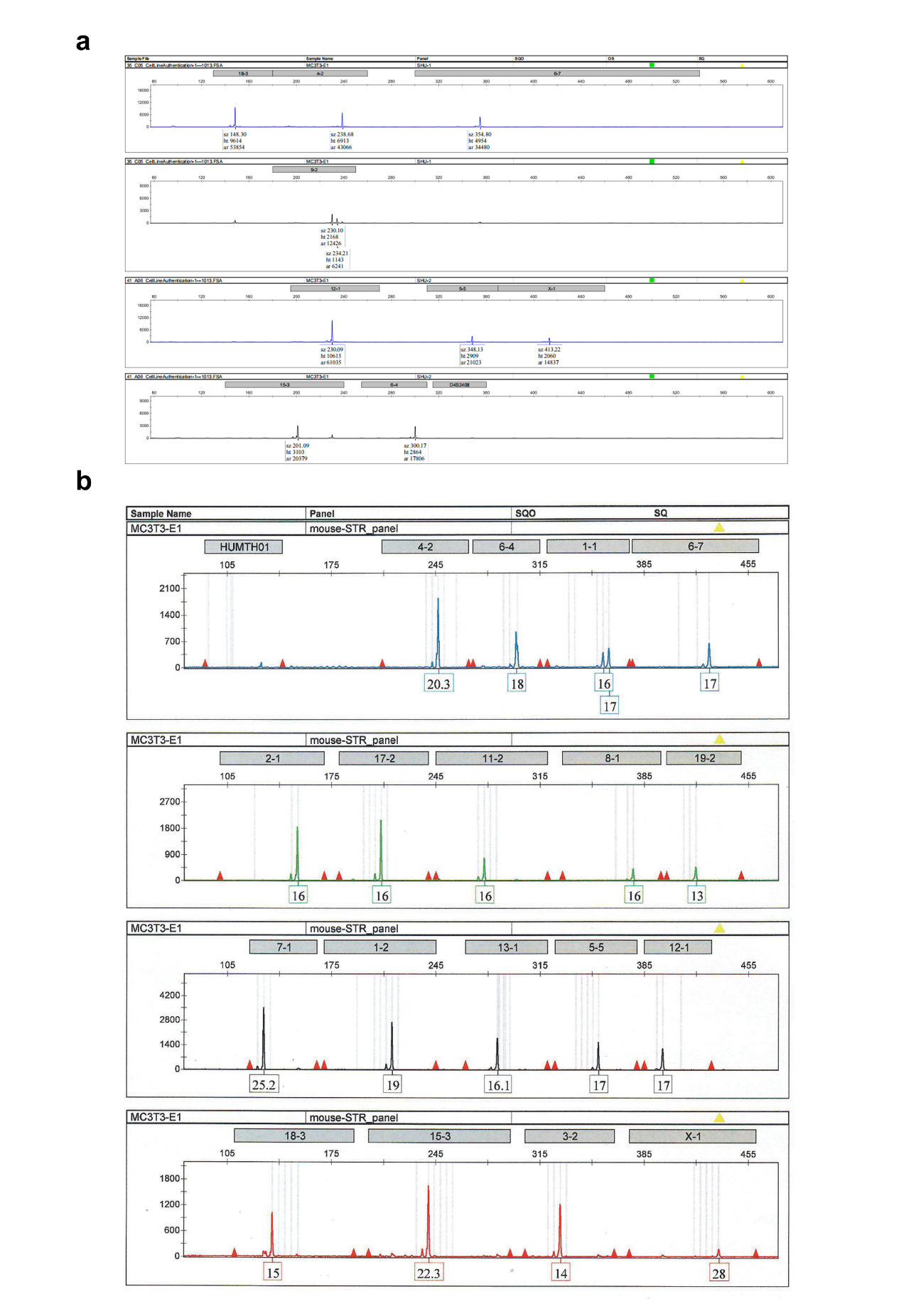


**Figure S2.** Identification of hDFSCs. a, Flow cytometry analysis. b, Chondrogenic differentiation, adipogenic differentiation, and osteogenic differentiation staining.


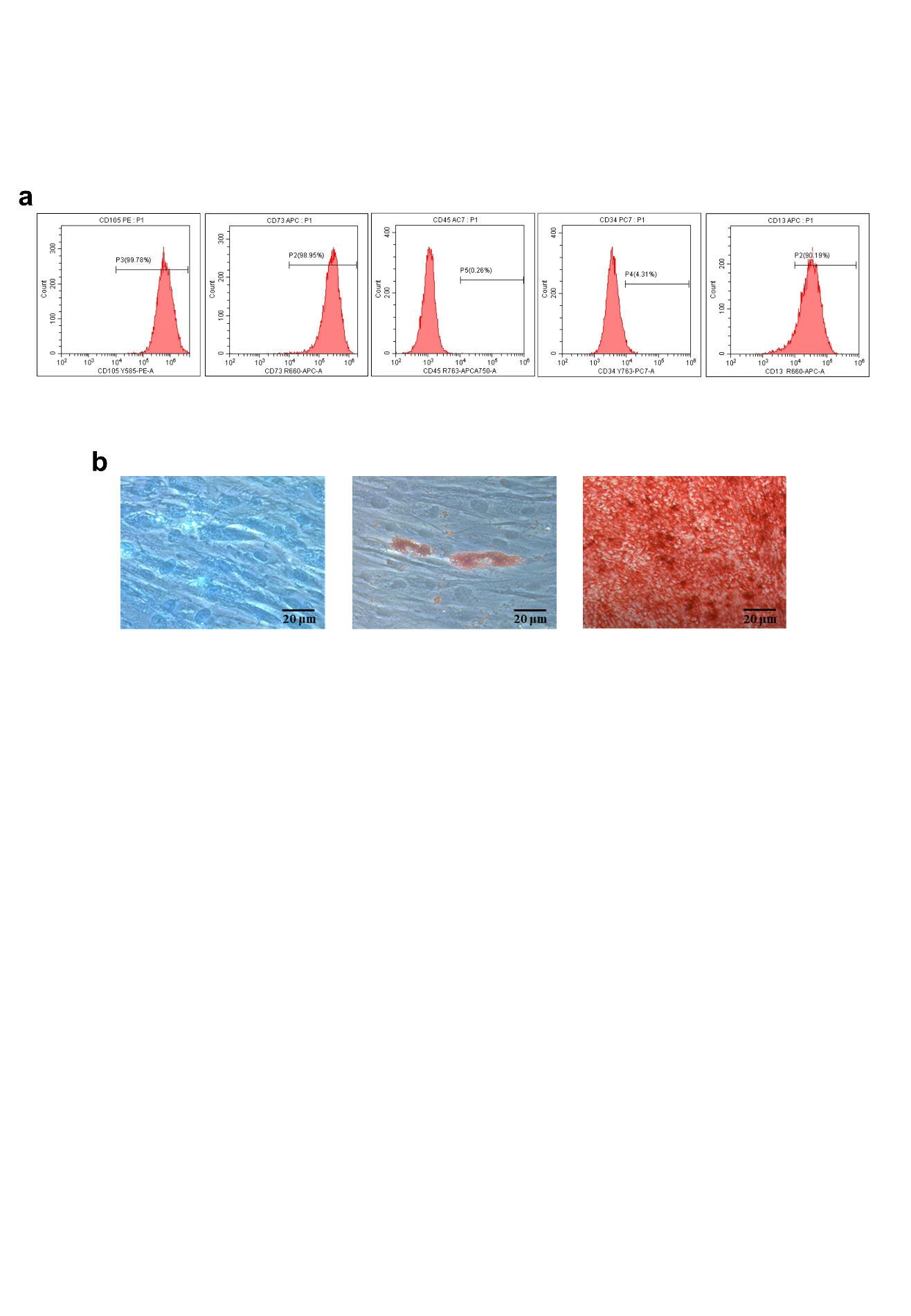


**Figure S3.** Organs’ distribution of DRDENs in C57BL/6 after caudal vein injection, intraperitoneal injection, and oral administration 48 hours. (From top to bottom and left to right: heart, liver, spleen, lung, kidney, and gastrointestinal tract).


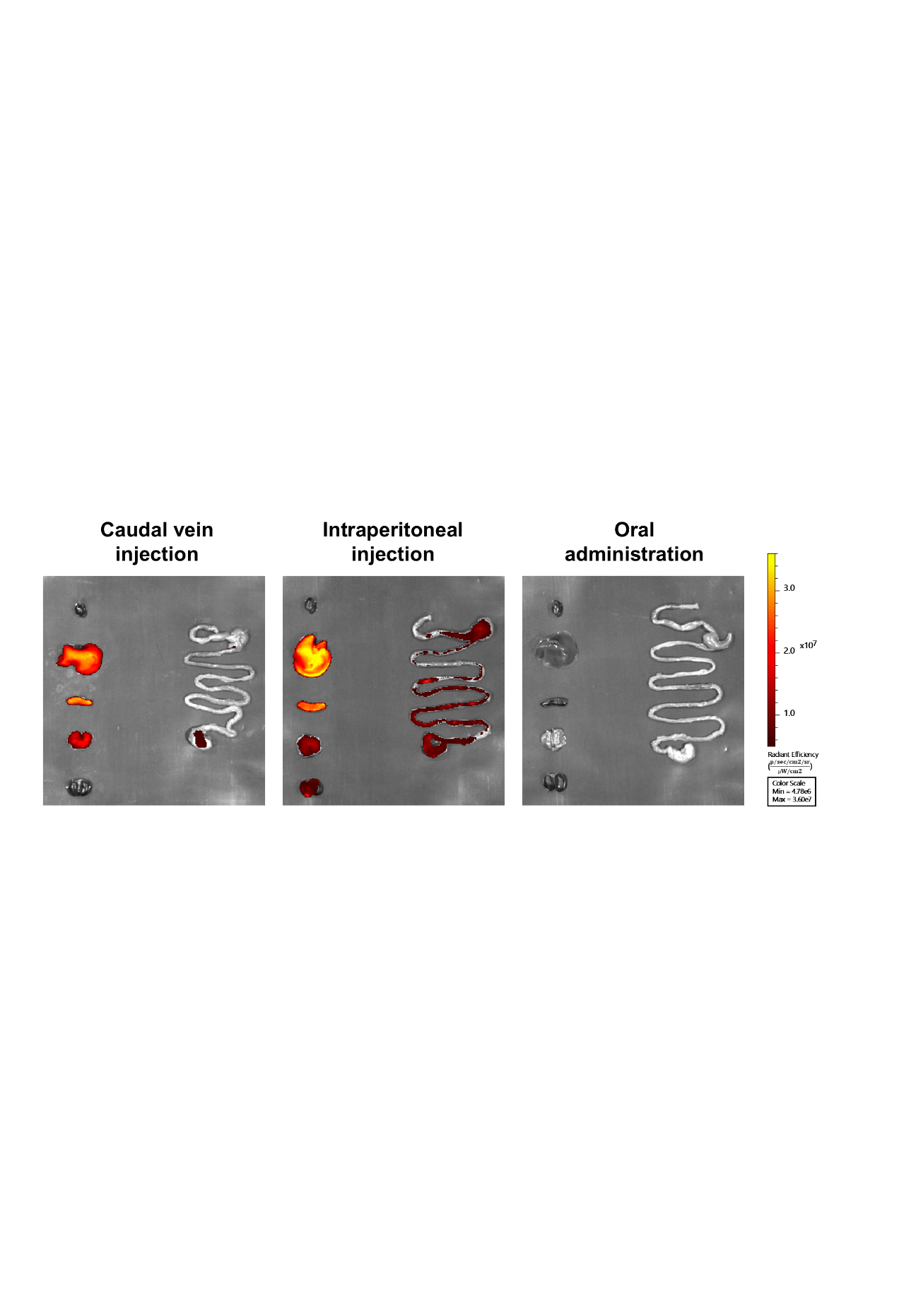


**Figure S4.** In vitro digestion assay of DRDENs in simulated gastric solutions, with a gradient pH from 1 to 3 for 1 h at 37 °C. a, TEM analysis of DRDENs after being digested with gastric acid simulation solution (pH 1). b, Size distribution and concentration change of DRDENs in gradient pH gastric acid simulation solution.


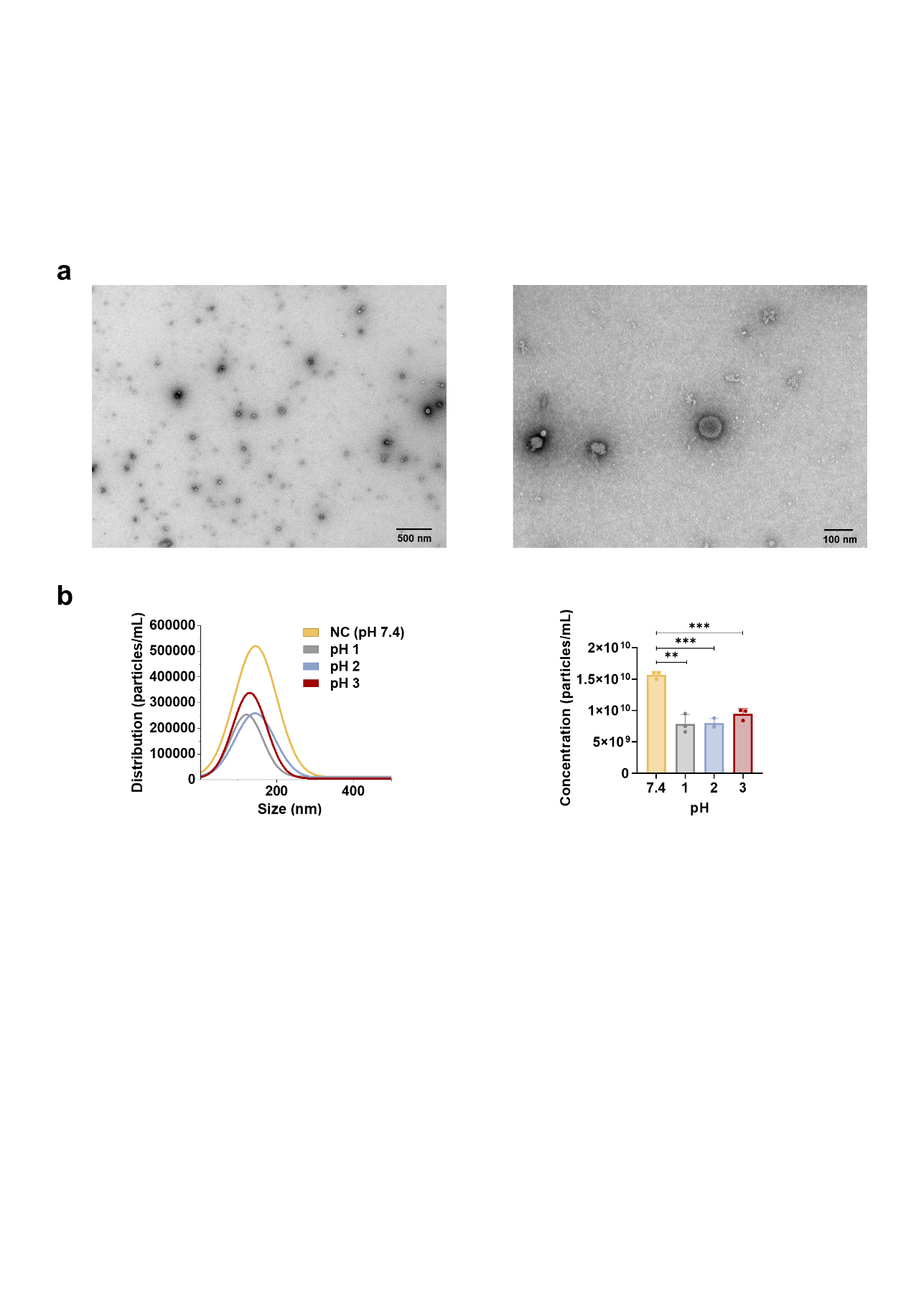


**Figure S5.** Molecular docking. a, Affinity of naringenin chalcone and IP3 with ITPR3. b, 2D plot of interaction force between naringenin chalcone and ITPR3.


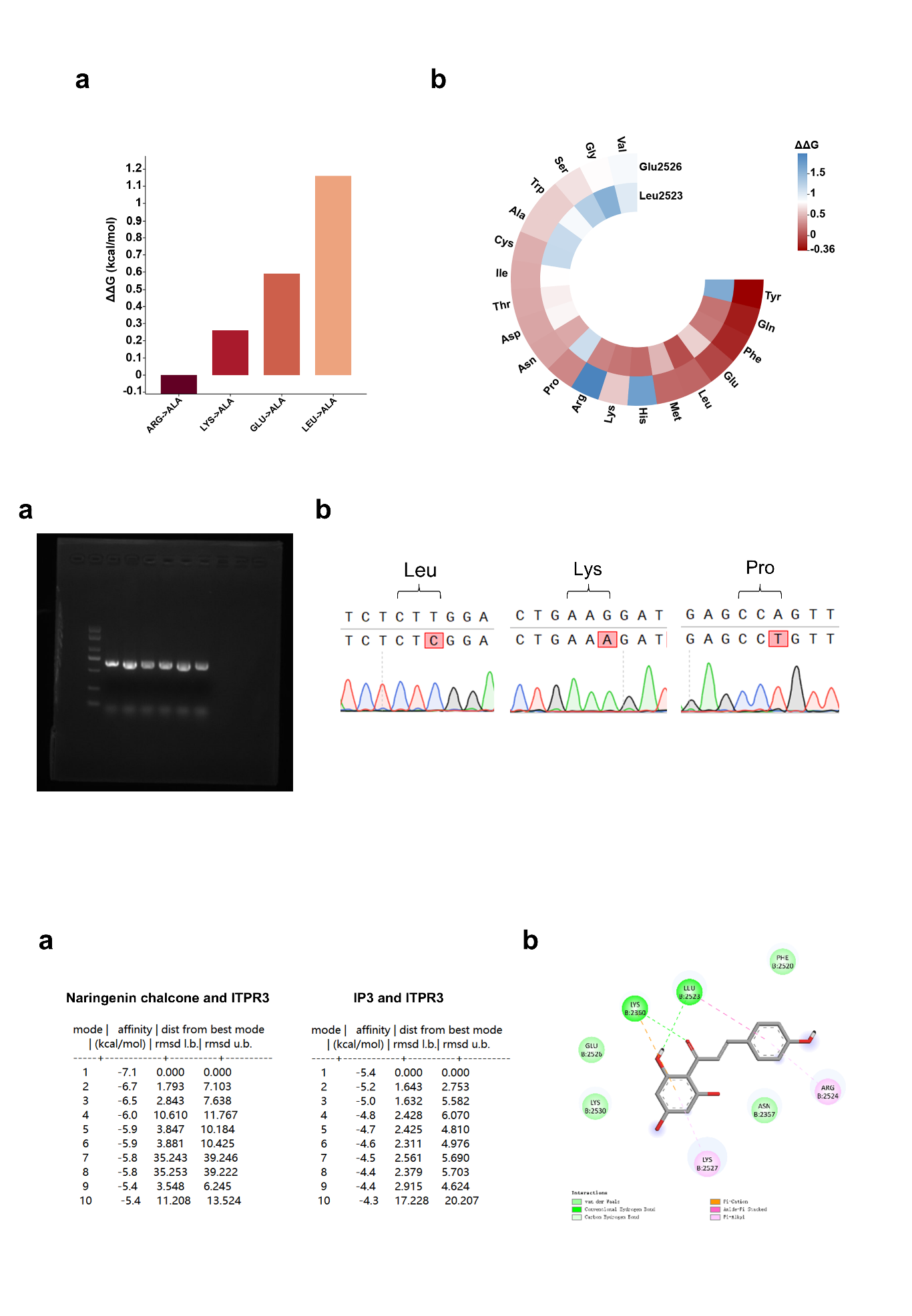


**Figure S6.** Molecular dynamics simulation. a, Root mean square deviation (RMSD) analysis. b, Root mean square fluctuation (RMSF) analysis. c, Radius of gyration (RoG) analysis. d, Solvent accessible surface area analysis (SASA). e, f, Decomposed and total binding free energy of residues.


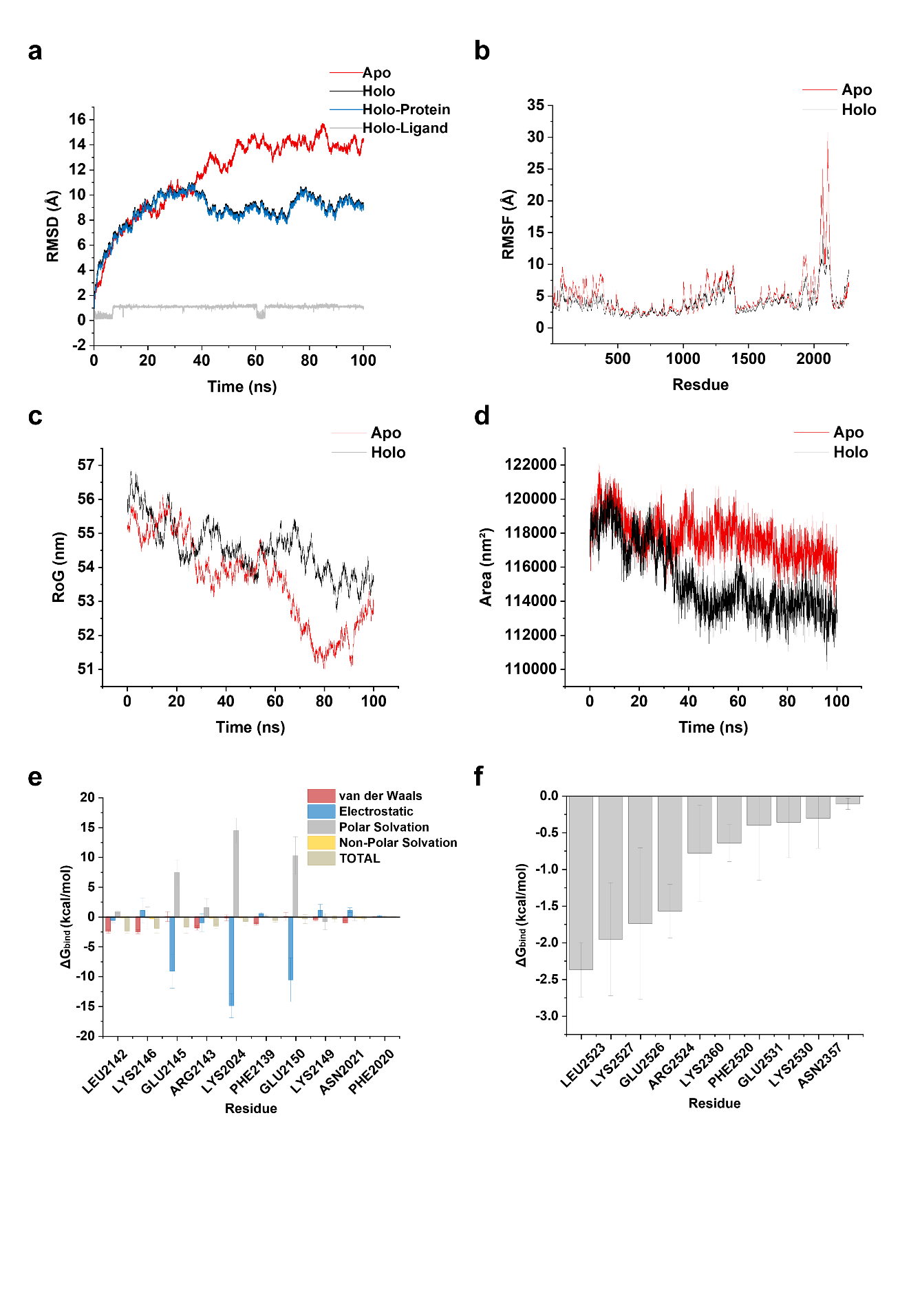


**Figure S7.** Virtual mutation of amino acid residues. a, Computational alanine scanning. b, Saturation mutagenesis of predicted key binding residues between naringenin chalcone and ITPR3.


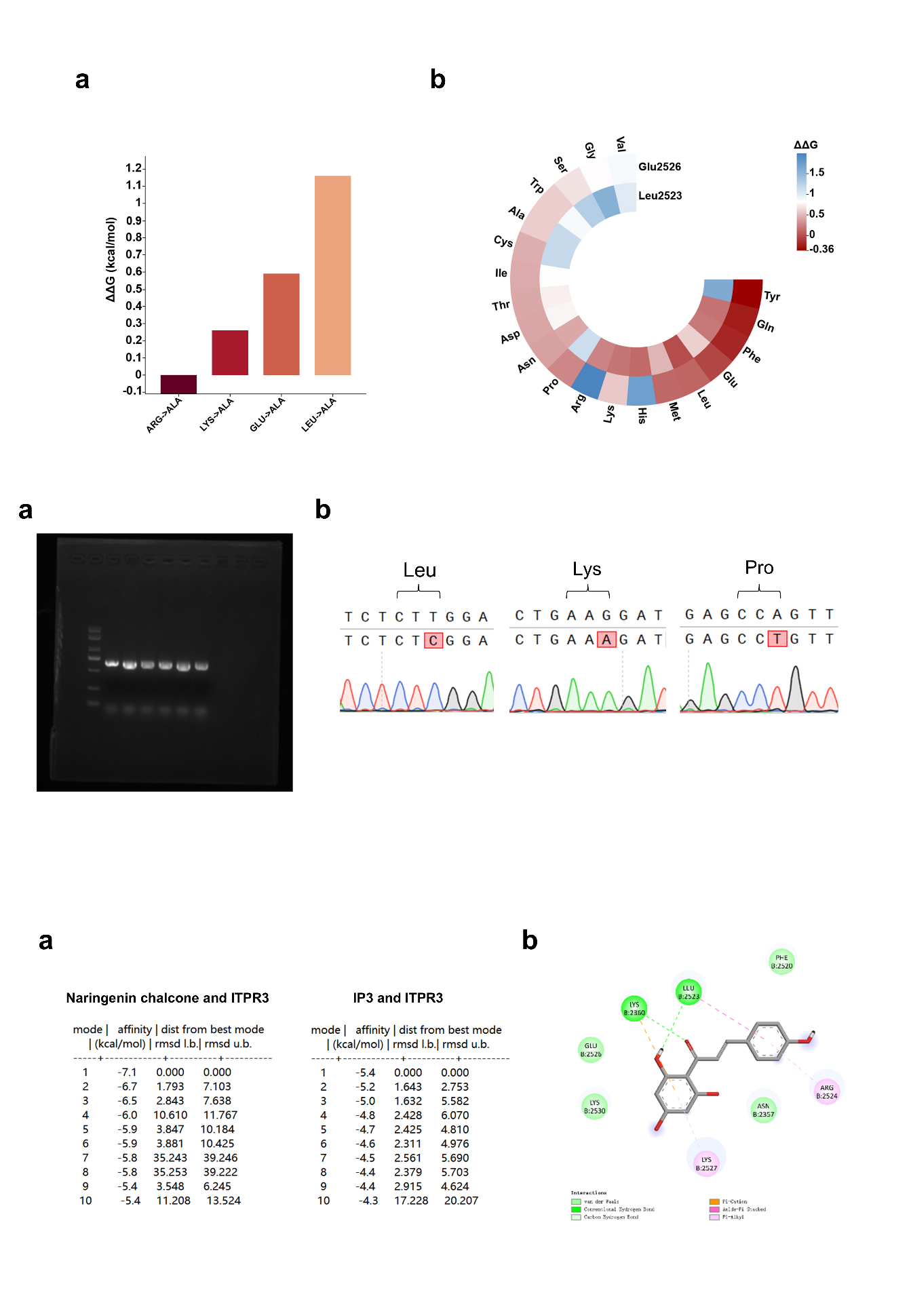


**Figure S8.** Standard curve establishment of naringenin chalcone using LC-MS.

**
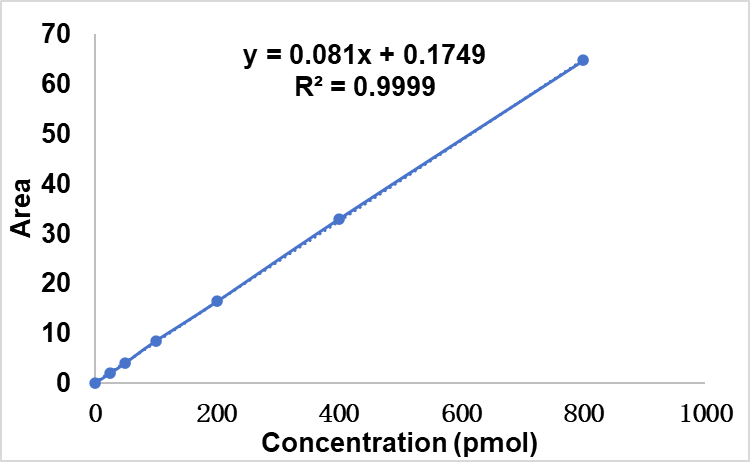
**

**Figure S9.** DNA fragment of *DrCHI*. a, DNA agarose electrophoresis of *DrCHI* (DNA marker from top to bottom: 2000, 1500, 1000, 750, 500, 250, 100 bp). b, DNA sequencing result of *DrCHI*.


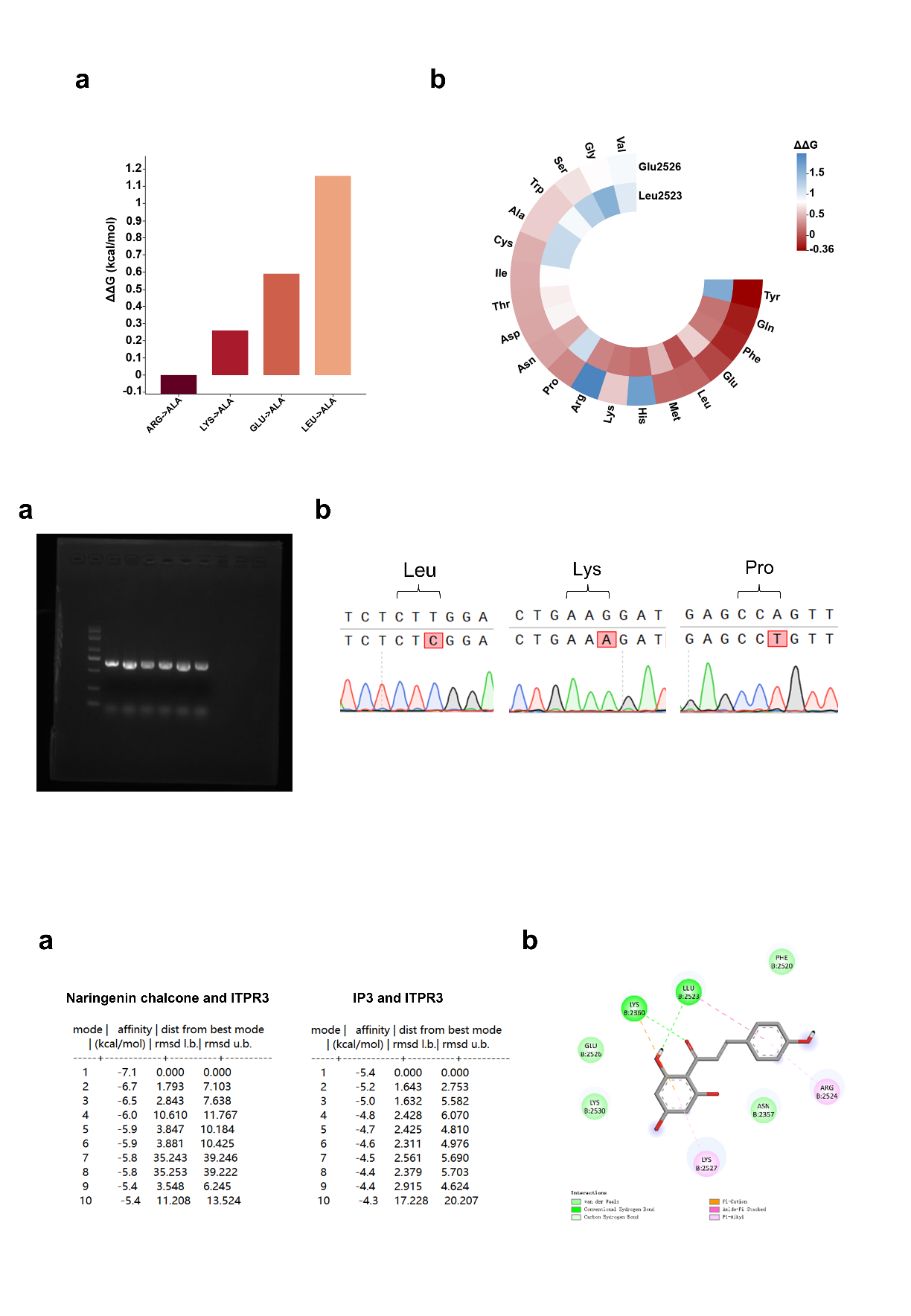


**2 Supplementary Tables**

**Table S1.** Primers for qRT-PCR.

| **Gene** | **Forward primer (5′-3′)** | **Reverse primer (5′-3′)** |
| --- | --- | --- |
| ALP (mouse) | TCATTCCCACGTTTTCACATTC | GTTGTTGTGAGCGTAATCTACC |
| RUNX2 (mouse) | CCTTCAAGGTTGTAGCCCTC | GGAGTAGTTCTCATCATTCCCG |
| OCN (mouse) | CTGCGCTCTGTCTCTCTGAC | TTAAGCTCACACTGCTCCCG |
| β-actin (mouse) | CTACCTCATGAAGATCCTGACC | CACAGCTTCTCTTTGATGTCAC |
| ALP (homo sapiens) | TAAGGACATCGCCTACCAGCTC | TCTTCCAGGTGTCAACGAGGT |
| RUNX2 (homo sapiens) | CTTTACTTACACCCCGCCAGTC | AGAGATATGGAGTGCTGCTGGTC |
| OCN (homo sapiens) | CTCACACTCCTCGCCCTATT | CCTCCTGCTTGGACACAAA |
| β-actin (homo sapiens) | CAGCCTTCCTTCCTGGGCATG | ATTGTGCTGGGTGCCAGGGCAG |

**Table S2.** Primers for DNA amplification and verification.

| **Gene** | **Forward primer (5′-3′)** | **Reverse primer (5′-3′)** |
| --- | --- | --- |
| *ITPR3* | TTCCCAGCCCGAGTGGTCTAT | AGGAAGGGCCAAACCGCTACT |
| *DrCHI* | CCGGAATTCATGGCACTCGCT  GCCCAGCAAAAG | CCGCTCGAGTCACATGTACTTG  GACAAACGCTCCGC |

**Table S3.** Binding free energy calculated by MM/GBSA.

| **System name** | **Protein/ligand (kcal/mol)** |
| --- | --- |
| ΔE_vdW_ | -23.75±2.72 |
| ΔE_elec_ | -60.65±11.96 |
| ΔG_GB_ | 64.61±10.02 |
| ΔG_SA_ | -4.43±0.36 |
| ΔG_bind_ | -24.21±2.59 |

ΔE_vdW_: van der Waals energy.

ΔE_elec_: electrostatic energy.

ΔG_GB_: electrostatic contribution to solvation.

ΔG_SA_: non-polar contribution to solvation.

ΔG_bind_: binding free energy.

**Table S4.** SPR result of ITPR3 and naringenin chalcone.

| **Protein** | **Ligand** | **K_a_ (1/Ms)** | **K_d_ (1/s)** | **K_D_ (M)** |
| --- | --- | --- | --- | --- |
| ITPR3 | Naringenin chalcone | 3.35e+04 | 1.47e-01 | 4.39e-06 |

**Table S5.** DNA and amino acid sequences of DrCHI.

| **DrCHI** | **Sequence** |
| --- | --- |
| DNA | atggcactcgctgcccagcaaaagagacagctggagttccatggcttggacatcgaaggtatcagctttgtgtcctcagcggtatctctcggatcctcaaagaaactggtcttgggtggtgctggcaatagaggccttgaaattaacggcaaatttgtaaagtttacagcaattggaatttatgtagaagatggaatcgtccgctatctctccccaaaattgggtggcaagtctgtggaggagctctgtgacaaagagttgctctttgaagaagtgctatcagctcctgttgagaaattagtgcgcgttgtttttcttcttcctctaactggtcctcagtattccgagaaagttttggagcgcatcggtgtgcaaggcttgtactcaaatctgaaagatgagcacaaaaatcagttcttggaaattttcaaagctgagaattttcctcctaggtcgtctttattcctttccttcaccgaaaaagggttgaaggttgcatttagcaaaggtgatgatattcctgaagagcctgttgcagctattgaggataaatcatttgcggatgcggtccttgcgaccatcatctggaaagatggagtgtcgccggcagcaaaagtatcgcttgcggagcgtttgtccaagtacatgtga |
| Amino acid | MALAAQQKRQLEFHGLDIEGISFVSSAVSLGSSKKLVLGGAGNRGLEINGKFVKFTAIGIYVEDGIVRYLSPKLGGKSVEELCDKELLFEEVLSAPVEKLVRVVFLLPLTGPQYSEKVLERIGVQGLYSNLKDEHKNQFLEIFKAENFPPRSSLFLSFTEKGLKVAFSKGDDIPEEPVAAIEDKSFADAVLATIIWKDGVSPAAKVSLAERLSKYM |

**Table S6.** Genbank accession number and sequences of 22 ferms CHIs used in multiple sequence alignment and phylogenetic analysis.

| **Fern** | **Genbank No.** | **Sequences of CHI** |
| --- | --- | --- |
| *Adiantum capillus-veneris* | QDF63006.1 | MTDAVENKQVEFGALEVEGIKFAPSVAALGSTKQLVLGGAGFR  GLEISGNLVKFTAIGIYVDEAIIPHLSPKLHGKSIEELCNNELLFEE  VLAAPFEKLVRVVFLLPLTGPQYSEKVVERMGLLPNPGIKEEST  KQFLEIFKPENFPPRTSLICSFTEEALKVAFMKTDDFPEDADAVI  EDKWLARAFLASIIGKDGVSPLAKLSFAERMSRCL |
| *Adiantum caudatum* | QDF63019.1 | MTGPVENKQLELGGLEVEGIEFAPSVAALGSSKQLVLGGAGFR  GLEINGNLVKFTAIGIYVDEAIIPHLSPKLHGKSVEELCENELLFE  EVLAAPFEKLVRVVFLLPLTGPQYSEKVVERMGLLPNLGLKEE  STKQFLEVFKHESFPPRTSLVCSFTEEALKIAFMKGDGFPKEP  DAVIEDKSLACAFLASIIGKDGVSPMAKLSFAERLSRYL |
| *Adiantum nelumboides* | MCO5552155.1 | MDANPTKSQAQCMLTSSLPLLSGGLENKQVELGALEVEGIEFA  PSVAALGSSKQLVLGGAGFRGLEINGNLVKFTAIGIYVDEAIISH  LSPKLHGKSVEELCENELLFEEVLAAPFEKLVRVVFLLPLTGPQ  YSEKVVERMGLLPNLGLKEESTKQFLEVFKHETFPPRTSLVCS  FTEEALKIAFMKGDGFPKEPDAVIEDRNLACAFLASIIGKDGVS  PLAKLSFAERMSRHL |
| *Aglaomorpha bonii* | QDF63017.1 | MALAAQQKRQLEFHGLDIEGISFESSAVSLGSSKKLVLGGAGN  RGLEINGKFVKFTAIGIYVEDGIVRYLSPKLGGKSVEELCDKEL  LFEEVLSAPVEKLVRVVFLLPLTGPQYSEKVLERIGVQGLYSNL  NDEHKKQFLEIFKAENFPPRSSLYLSFSDKGLKVAFSKGDDIPE  EPVAAIEDKLFADAVLATIIWRDGVSPAAKVSLAERLSKYM |
| *Antrophyum callifolium* | QDF63009.1 | MGSEVVSKKHEFGALEVEGIDFRASVAALGSTKKLVLGGAGFR  GLEINGNLVKFTAIGIYVEEAIIPHLSPKLGGKTVEELCANELLFE  EVLSAPFDKLIRVVFLLPLTGPQYSEKVVERIGLLPNPGLKEESI  KQFLEIFKAENFPPRTSLVCSFTEDGLKIAFMKGPDFPEESDAV  IEDKYFARAFLATIIAKDGVSPMAKLSFAERVSKYL |
| *Arthropteris palisotii* | QDF58101.1 | MMLTAQQERQMEFNGLDIDGISFISSVVAFGSSKALVLGGAGD  RGIEINGKFIKFTAIGIYVDDGIISHLSPKLGGKSVEELCDKELLF  EEIVSAPFEKLVRVAFLVPLTGPQYSEKVLEQIGAQGMYRDLKD  EHKQQFLEIFKAESFPPMSSIMFSFTKEGLKMAFVQGNEIPEKP  VAVVEDKMFADAVLASIIGKGGVSPAAKVSLAERLSKYL |
| *Asplenium loriceum* | QDF63011.1 | MGLSAQQAKRMEFHGMDIEGSRFEPSVVAPGSSKALVLGGAG  DRGLEIHGKFIKFTAIGIYVEDAVIPYLSPKLGGKSVEELCEKELL  FEEVLSAPFEKLVRVVFLLPLSGPQYSEKVLERIGVQALYTDVK  DEHKQQFLEIFKAQNFPPRSSLVLSFTETGLKVAFPKGDDILEK  PAAVIEDKTFADAVLATIIWKDGVSPAAKVSLAERLSEYF |
| *Ceratopteris richardii* | KAH7285587.1 | MSAFSTMTNACGCKQLDFGSLDIEGIPFAATATTLGSTTELILG  GAGFRGLEIQGKLIKFTAIGIYVEAAIIPHLSQKLAGKSLEELCEN  DLIFNEVVAAPHDKLVRVTFLAPLTGPQYSEKVVERIGLIENSGV  KEESLKLFLEIFKTENFPPGTSVVVSFTKSALKIAFTKDNEIPKE  PAAVIEDEAFACGFLASIIGKDGVSPAAKNSFAKRIYNHLK |
| *Cibotium barometz* | QDF63014.1 | MAILEHQEKAHDFDGLEVEGVAFAPSIVAPGSSKPLILGGAGDR  GLEINGNFIKFTAIGIYVEEGVIPHLCPKLGGKTVAELCEKELLFE  ELLSAPFEKFVRVVFLVPLSGPQYSEKVLERIGAQALYTKLQDE  HKQQFLEIFKAENFPPRSSVLFSFSKEGLKVAFTKGNNIPEKPV  AVIKDETFAEAVLATIIWKEGVSPGAKVSLAERLSKCF |
| *Dicksonia antarctica* | QDF63016.1 | MASLDQQERVHDFEALEVEGVPFAPSIVAPGSSKALVLGGAGD  RGLEINGNYIKFTAIGIYVEEGVIPHLSPKLSGKTVEELCDKELLF  EELLSAPFEKFVRVVFLVPLSGPQYSEKVLERIGVQALYTELQD  EHKQQFLEIFKAESFPPRSSVLLSFSKEGLKVAFTKGNDIPEKP  VAVIEDETFGEAVLATIIWKEGVSPAAKVSLAERLSKYF |
| *Goniophlebium niponicum* | QDF63024.1 | MRRLENYLYERGWLALPLFKEGREAVLLPHRTSFPGASARSN  PSATTMALAAQQKRQLEFHGLDIEGISFESSAVSLGSSKKLVL  GGAGNRGLEINGKFVKFTAIGIYVEDAIVHYLSPKLGGKSVEEL  CDKELLFEEVLTAPVEKLVRVVFLLPLTGPQYSEKVIERIGVQG  MYPNLKDEYKKQFLEIFKAENFPPQSSLFLSFTKKGLKVAFSK  GDGFPEQPVAAIEDKSFADAVLATIIWKDGVSPAAKVSLAERL  SEYM |
| *Haplopteris amboinensis* | QDF63010.1 | MAYGAESKKEGFGALDVEGIDFASSVAALGSSKELVLGGAGFR  GLEINGNFVKFTAIGIYVEEGIVSHLSSKLGGKSVEELCENELLF  EEVLSAPFDKLVRVVFLLPLTGPQYSEKVVERMGLLPNLKIKEE  STKQFLEIFKPENFPPRTSLVCSFTEHGLKIAFMKGSDFPKEPD  AVIEDKHFARAFLATIIGKDGVSPMAKLSFAERVQKYL |
| *Hypolepis punctata* | QDF63015.1 | MADIAPTETQWEFSGLDVEGIAFGASVTGLGSSKALILGGAGD  RGLEINGNYIKFTAIGIYVEDGIIPHLSPKLNGKSVEELCDNELLF  EEVLSAPFEKLVRVVFLLPLSGPQYSEKVLERIGVQAMYTDVK  DEHKQQFLELFKPENFPPRSSVIFSFTKEGLKVAFTKGNDIPEK  PVAVIEDKTFADAVLATIIWKEGVSPAAKVSLAERLSKYL |
| *Lindsaea orbiculata* | QDF63001.1 | MTFEEKPMKFDGLDIEGVAFAPSVAALGSAKPLVLGGAGDRGL  EINGNYIKFTAIGIYIEDGIIPHLSSKLSGKTVLELCEKDLLFEEVI  YAPFEKLVRVVFIAPLTGIQYSEKVLERISVQALYTDIKEEHKQQ  FLEIFKGESFPPQSSAILSFSKEGLKVAFSKGDDVPAKAVGTIEN  ARFADAVLGTIIWRDGVSPAAKVSLAERVSKYLQLGPSDGLAS  VPQSK |
| *Lomagramma matthewii* | QDF58100.1 | MPVVLSAQQDKEFKFDGLNIEGISFESSVRALASTKMLVLGGA  GDRGLEKNGKFIKFTTIGIYVDDAIIPDLSRKLGGKTVDELCDKE  LLFEEVVAAPFEKLLLIYFLLPLTGPEYAEKVFERIDVQGIYKDLK  EESKQNFEGIFAAEHFPPGSCLVLSFSAEGLKVAFTKVGEKMP  EKPVAVVEDRRFADAYLTTIICKDGVSPSAKLSLAERLSKYF |
| *Matteuccia struthiopteris* | QDF63025.1 | MALAAQQEKQMEFHGLDVEGITFEASVVALGSSKTLVLGGAG  DRGLEINGRVIKFTAIGVYVEDGIVPYLSPKLSGKSVEELCDKE  LLFEEVLSAPFEKLVRVFFLLPLTGPQYSEKVLERIGVQAIYTDL  KDEHKQQFLEIFKAENFPPRSSLVFSFTEEGLKVAFPKGNDIPE  KPVAVIEDKAFADAVLATIIWKDGVSPAAKVSLAERLSKYF |
| *Microlepia speluncae* | QDF63020.1 | MAPKQWEFGGLDIEGVAFAPSVKGLGCSNSLILGGAGDRGLE  INGNYIKFTAIGIYVEEGIIPHLSPKLSGKSVEELCDKELLFEEVL  AAPFEKLVRVVFLLPLSGPQYSEKVLERIGVQAMYTDLKDEHK  QQFLEVFNPENFPPRSSVILSFTKEGLKVAFTKGNEIPEEPVAV  IADKAFADAVLTTIIWKDGVSPAAKVSLAERLSKYL |
| *Odontosoria chusana* | QDF63000.1 | MTLDEKPRKFDGLDIEGVAFAASVTALGAAKPLALGGAGDRG  LEINGNYIKFTAIGIYVEDGIIPHLSPKLRGKTVEELCEKELLFEE  VISAPFEKLVRVVFLVPLTGIQYSEKVLERISVQSLFTDIKEEHK  QQFLEIFKGESFPPRSSVILSFSKEGLKVAFAKGDDVPSKAVGT  IEDATFADAVLATIIWKEGVSPAAKVSLAERLSKCL |
| *Pteris vittata* | QDF63013.1 | MSSVAEKTQWDFGGLEIEGVAFAPSVVAPGSTTPLILGGAGFR  GLEINGNLIKFTVIGIYVEDKIIPYLSPKLGGKSVAELCENELLFD  EVVAAPIEKLVRVTFLVPLTGPQYSEKVVERIGLLPNSGVKEES  TTQFLEIFKAESFPPRTSIVLSFSDKLLKIAYTQDNEIPKEPAAVI  EDEYFARAFLATIIGKDGVSPAAKLSFAERVSKKL |
| *Stenochlaena palustris* | QDF63021.1 | MALAAQQEKQREFLSLEIEGMTFGSSVVALGSSKMLVLGGAG  DRGLEINGRFIKFTAIGIYIEDGIIPYLSPKLSGKSVEELCHKELLF  EEVLSAPFEKLVRVVFLLPLTGPQYSEKVLERIGVQGIYKDLRE  EHKQQFLEIFKAETFPPRSSLVFSFTEEGLKVGFPKGNEIPEEP  VAVIPNKSFADAVLATIIWKDGVSPAAKVSLAERLSKYF |
| *Thelypteris parasitica* | QDF63008.1 | MALVAQQEKLRELHGLDIEGIIFEPSVQALGSSKSLVLGGAGD  RGLEINGKFIKFTAIGIYVEDGIVPYLSPKLSGKCVEELCDKELL  FEEVISAPFEKLVRVVFLLPLTGPQYSEKVLERIGVQGMYKDLK  DEHKQKFLEIFKAESFPPRSSLELSFTEQGLKVAFPKGNDIPEK  PVAVIEDKTFADAVLTTIIWKDGVSPAAKVSLAERLSKNF |
| *Goniophlebium amoenum* | QDF63005.1 | MALAAQQEKHMEFHSLEIEGMTFESSVVALGSSKVLVLGGAG  DRGLEINSRFIKFTAIGIYVEDGIVPYLSPKLGGKSVEELCDKEL  LFEEVLSAPFEKLVRVVFLLPLTGPQYSEKVLERIGVQGIYKEL  KDEHKQQFLEIFKAENFPPRSSLVFSFTEEGLKIAFPKGNEIPE  KPVAVVADKAFADAVLATIIWKDGVSPAAKVSLAERLSKYF |

**Table S7.** Plasmids, strains, and cells used in this study.

| **Strain/Plasmid** | **Purpose** |
| --- | --- |
| **Plasmid** |  |
| pET28a | Protein expression vector |
| pGenlenti-U6-ITPR3(human)-sgRNA-Cas9 | Gene editing |
| pAAV-ITPR3-Donor | Gene editing |
| pSPAX2 | Lentivirus packaging |
| pMD2.G | Lentivirus packaging |
| **Strain** |  |
| *E. coli* DH5α | Gene cloning |
| *E. coli* Rosetta | Protein expression |
| **Cell** |  |
| MC3T3-E1 cells | Cytotoxicity, osteogenesis |
| hDFSCs | Cytotoxicity, osteogenesis, and cellular mechanism |
| HEK293T | Lentivirus packaging |
